# Efficient Photocatalytic Degradation of Textile Dyes Using Four-Element Doped Anatase Nanocrystals under Low-Intensity LED Light

**DOI:** 10.1021/acsomega.6c00166

**Published:** 2026-06-02

**Authors:** Yahya Absalan, Abhyuday Mandal, Ramaraja Pandian Ramasamy, Baviththira Suganthan, Yehia Khalifa, Suraj Sharma

**Affiliations:** † Department of Chemistry, 1355 University of Georgia, Athens, Georgia 30602, United States; ‡ Department of Statistics, University of Georgia, Athens, Georgia 30602, United States; § Nano Electrochemistry Laboratory, School of Chemical, Materials and Biomedical Engineering, College of Engineering, University of Georgia, Athens, Georgia 30602, United States; ∥ College of Arts and Sciences, Department of Chemistry and Biochemistry, 2647 The Ohio State University, Columbus, Ohio 43210, United States; ⊥ Department of Textiles, Merchandising, and Interiors, University of Georgia, Athens, Georgia 30602, United States

## Abstract

An essential challenge in environmental catalysis is the development of efficient, visible‑light–active photocatalysts. In this study, we report the synthesis of seven anatase-phase nanocrystals doped with different dopants, including constant dopants of cerium (Ce), nitrogen (N), and and various alkali/alkaline‑earth metals.. Among the compositions studied, the strontium (Sr)-doped nanocrystal (NP­(Sr)) exhibits superior photocatalytic activity, completely degrading cationic, anionic, reactive, and azo textile dyes in less than 1 h under ultra-low-intensity light-emitting diode (LED) illumination (9.55 mW cm^–2^). This achievement is attributed to lattice strain-induced surface activity, reduced crystallite size, and enhanced charge transport. The high active surface area obtained through anisotropic growth along the {001} plane by ethylenediamine results in a fast light response. The dominant role of superoxide radical generation and synergistic tuning of the band structure by Ce and N, confirmed by Mott–Schottky, cyclic voltammetry, photoluminescence, and electron paramagnetic resonance (EPR) spectroscopy, provided mechanistic insight into dye degradation. Furthermore, the localization of nitrogen within the bulk lattice and its high stability over multiple cycles demonstrate a scalable, low-energy solution for industrial wastewater treatment.

## Graphical abstract

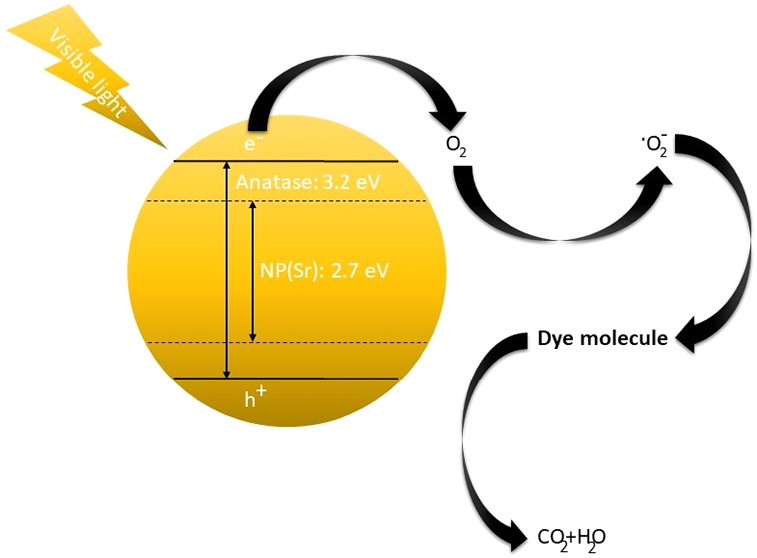

## Introduction

The applications of textile dyes span multiple industries, including the textile industry, food industry, leather industry, pharmaceutical industry, and technology industry.^1–4^ However, the harmful effects of textile dyes, including histopathological changes in animals, the environmental impact of dye discharge, toxicity, allergic reactions, and the need for upcycling wastewater, are widely recognized.^5–9^ These issues and many others make textile dyes the second most harmful pollutant after oil.^10^ The quest to remove textile dyes from water has led to the development of diverse methods. These can be broadly categorized into three main groups: physical, chemical, and biological. Each method has its unique approach and benefits. However, each of these methods has its disadvantages. Overall, neither of these methods can completely remove textile dyes, and they almost always require further treatment. Additionally, byproducts are another concern, particularly with the chemical and biological methods used in water treatment. The cost associated with precursors and facilities can be significant. Additionally, in chemical and biological processes, the use of certain harmful compounds often leads to further issues.^11–17^

Photocatalytic degradation is an effective method for removing textile dyes from water. This method offers several advantages, including being eco-friendly and sustainable, highly efficient, and versatile, with enhanced biodegradability, cost-effectiveness, and reduced toxicity. These features make it a compelling solution for removing textile dyes.^18–23^ Metal oxide nanocrystals have been utilized as promising photocatalysts due to their semiconductivity properties, which can be easily tailored and modified to absorb sunlight. Pristine TiO_2_ is not active under visible light due to its wide band gap, which is around 3.2 eV, and it limits its excitation to the UV region. These materials offer numerous advantages, including high photocatalytic efficiency, enhanced light absorption, improved stability and reusability, synergistic effects with other materials, versatility in treating various dyes, and being eco-friendly and cost-effective.^24–28^

However, some challenges make them less universal and less applicable on a large scale, such as wavelength, charge carrier recombination, degradation efficiency, charge separation, interfacial charge transfer inhibition, photocatalyst materials, and varying pH levels in solutions.^29^ Except for energy band gap tuning, almost all of the mentioned issues remain challenging in the first steps. In addition, most tuned photocatalysts, which are modified to absorb visible wavelengths, can operate under intense light such as a xenon lamp. Xenon lamps are the nearest example of sunlight intensity because of their high intensity. It produces high temperatures, requires more expensive facilities, and is less safe than light-emitting diode (LED) lamps. If a photocatalyst functions with an LED of low intensity, it will likely perform even better under sunlight.^30^ The synthesis of a photocatalyst that can effectively absorb visible light, even at low intensities, is a significant challenge.^31^ The ideal photocatalyst needs to demonstrate a strong response to light, a low electron–hole recombination rate, and also an appropriate band gap for efficient charge separation. Additionally, achieving a high efficiency across a range of varying pH levels is crucial.

Alkali and alkaline-earth elements are rarely used as dopants compared to transition metals. Transitional metals can exist in various oxidation states, enabling them to exhibit distinct properties, particularly in terms of absorbing visible wavelengths. Alkali and alkaline-earth metals cannot absorb visible wavelengths as effectively as transition metals. However, they are highly effective at enhancing charge carrier mobility, creating oxygen vacancies, and modifying the electronic properties, all of which play a vital role.^32–35^ Besides these advantages, they have significantly lower unpleasant impacts compared to heavy metals when they enter the water. They have a minimum risk when entering the water and for human health.^36^ Transition metals, such as chromium or cobalt, can accumulate in the food chain, leading to long-term environmental risks. In contrast, alkali and alkaline-earth metals can naturally degrade in the environment.^37^

The recent emphasis in the papers published recently demonstrates that, besides catalytic efficiency, mechanistic understanding and broad application are also essential. Recently, environmental photocatalysis has become a topic of growing interest, with numerous papers being published. Therefore, it is necessary to clearly study technological or scientific breakthroughs, such as visible light activity under realistic conditions or fundamental insights into charge dynamics. Many reports still rely on high-intensity UV or xenon lamps, achieving a limited scope across different dye classes.^38^

In this context, while Ce–N codoped and alkali/alkaline-earth metal doped TiO_2_ systems have been independently reported, the specific alkali/alkaline-earth–Ce–N combination and its optimization for ultralow light intensity operation (9.55 mW cm^–2^) have not been systematically explored. We demonstrate that this particular multidopant formulation achieves complete dye degradation under LED illumination intensities 10–100 times lower than typically employed, addressing a critical gap in practical photocatalyst development. This achievement significantly distinguishes this photocatalyst from traditional photocatalysts and meets key criteria for environmental relevance and energy efficiency. In addition, we conducted an optimization across 81 reaction conditions. We performed detailed investigations using electron paramagnetic resonance (EPR), cyclic voltammetry, and photoluminescence analysis to illustrate the role of each dopant in controlling the band gap, charge separation, and interfacial activity.

Furthermore, we explored the underlying mechanism using different theoretical and practical methods, which provided a comprehensive understanding of the superior performance of the photocatalysts. This multidopant approach differs from the standard single-doping method, as it utilizes f-block (Ce), s-block (Sr), and p-block (N) elements to alter the red shift of the band gap, enhance surface activity through lattice distortion, and suppress electron–hole recombination. These synergistic effects, when combined in the present system, enable complete dye degradation under ultralow LED intensity (9.55 mW cm^–2^), which is an order of magnitude below that commonly reported in photocatalysis studies. Therefore, benchmarking against pristine and commercial TiO_2_ is essential to prove the enhancement achieved through multielement doping under low-intensity LED illumination. In this work, we included commercial Degussa P25 TiO_2_ as a reference photocatalyst under the same experimental conditions to perform the practical performance gain of the proposed system.

## Experimental Section

### Material Characterization

The powder diffractometer Bruker D2 Phaser performed X-ray diffraction (XRD) analysis in the range of 10–70° using a zero-background holder operating with λ = 1.541 Å (Cu Kα). The crystallite size of the photocatalysts was determined using the Scherrer equation based on the XRD results. The morphology of the surface was examined using Field Emission Scanning Electron Microscopy (FESEM) (Thermo Fisher Scientific (FEI) Teneo), which was accompanied by energy-dispersive X-ray spectroscopy (EDS) to determine the composition of the photocatalysts and corresponding elements. Scanning Transmission Electron Microscopy (STEM) (Hitachi SU9000EA) was used to study the crystallography of the samples, accompanied by bright-field (BF) and High-Angle Annular Dark Field (HAADF) imaging. JEOL 2100PLUS was used to take the high-resolution transmission electron microscopy (HRTEM) image for studying the interplanar spacing of the adjacent lattice. To investigate the surface state of the photocatalysts, the Fourier transform infrared spectroscopy (FT-IR) spectrum was obtained in the mid-infrared range (4000–500 cm^–1^) using a Nicolet Nexus 650. The reflectance of the photocatalysts was measured using diffuse reflectance spectroscopy (DRS) with a PerkinElmer UV–vis Lambda 365 spectrometer in the range of 200–800 nm. The energy band gap was obtained by calculating the reflectance using a Tauc plot. Photoluminescence analysis (PL CARY ECLIPSE) measured the charge carrier recombination. Electron paramagnetic resonance (EPR) was performed using an EMXplus spectrometer to detect the radicals produced during the photocatalytic reaction. To study the surface redox behavior and charge-transfer characteristics of the samples, cyclic voltammetry (CV) was used through an Electrochemical Analyzer (CHI401A) from CH Instruments Inc., Austin, TX. The same machine was used to determine the conduction band through the Mott–Schottky (MS) method. Photocurrent spectroscopy was used to detect the photocatalyst’s response to the emitted light using a Metrohm DropSens μStat-i 400s. X-ray photoelectron spectroscopy (XPS) spectra were collected using a Thermo Fisher Scientific Nexsa G2 XPS system, which employed an Al Kα source and a flood gun. Samples were floated from the ground. All spectra were calibrated to C 1s at 284.8 eV. Survey spectra were collected at 100 pass energies, while high-resolution spectra were collected at 20 eV. The surface area of the photocatalysts was determined using the Brunauer–Emmett–Teller (BET) method, and the microporosity and pore size distribution were analyzed using the Barrett–Joyner–Halenda (BJH) method via a micrometric Belsorp-mini II. The elemental analysis was done with CHN and Inductively Coupled Plasma (ICP) through Thermo Finnigan (FLASH EA 1112 SERIES) and ICP-optical emission spectroscopy (ICP-OES), respectively. The ζ potential was measured on both bare TiO_2_ and NP­(Sr) to investigate the surface charge behaviors and their relationship with dye adsorption using a Malvern Zetasizer Nano ZS at 25 °C. The basic and acidic environments were obtained using 0.01 M NaOH and HCl. The solvent was water, and the ζ run was 100.

### Materials and Reagents

All of the materials, including titanium butoxide, chloride salts of cesium, calcium, strontium, barium, sodium, magnesium, rubidium, cerium, ethylenediamine (Sigma-Aldrich), 1-butanol, dimethylformamide (DMF), NaOH, HCl, and acetic acid (Fischer), were used without further purification. Disposable carbon screen-printed electrode (SPE) TE100 was acquired from CH Instruments, Inc. (Austin, TX) and used as working, counter, and quasi-reference electrodes.

### Synthesis of Photocatalysts

In the first step, 0.25 mol of titanium butoxide was dissolved in 50 mL of 1-butanol (15 min stirring; solution A). Then, 0.04 mol of the solution of “M” (alkali or alkaline-earth metals) and 0.01 mol of cerium chloride were mixed in 1 mL of deionized (DI) water (solution B). In the next step, solution B was slowly added to solution A. The solution was mixed for 30 min. After that, 0.07 mol of ethylenediamine was added slowly (due to the amine group and increasing pH, the precipitate was observed). The solution was mixed for 120 min to form a gel, ensuring the beaker was covered with aluminum foil. Then, the gel was placed in a hydrothermal autoclave for 18 h at 120 °C. Afterward, the solution was washed and centrifuged three times with ethanol and water at 7000 rpm. Then, the obtained product was dried at 60 °C for 24 h. Finally, the obtained powder was ground and calcined at 450 °C for 45 min at a rate of 10 °C min^–1^.

### Notation and Dopant Incorporation

The notation used throughout this work is summarized below. In all samples, cerium (Ce) and the alkali/alkaline-earth metal dopants (M = Cs, Ca, Sr, Ba, Na, Mg, and Rb) are substituted at Ti^4+^ lattice sites (substitutional doping). Nitrogen is incorporated interstitially between Ti and O atoms, as confirmed by Fourier transform infrared (FT-IR) and XPS analyses. The different valence substitution induces oxygen vacancies for charge compensation. Accordingly, the general chemical composition of the photocatalysts can be expressed as

$${\mathrm{Ti}}_{1-x-y}M_{x}{\mathrm{Ce}}_{y}O_{2-\delta }N_{z}$$

where *x* represents the fraction of alkali/alkaline-earth metal dopant (M), *y* denotes the Ce substitution fraction, *z* is the amount of interstitial nitrogen, and δ represents oxygen vacancies.

The specific notation used for each synthesized sample is summarized as follows:

$$\mathrm{NP}\left(\mathrm{Cs}\right):{\mathrm{Ti}}_{1-x-y}{\mathrm{Cs}}_{x}{\mathrm{Ce}}_{y}O_{2-\delta }N_{z}$$

$$\mathrm{NP}\left(\mathrm{Ca}\right):{\mathrm{Ti}}_{1-x-y}{\mathrm{Ca}}_{x}{\mathrm{Ce}}_{y}O_{2-\delta }N_{z}$$

$$\mathrm{NP}\left(\mathrm{Sr}\right):{\mathrm{Ti}}_{1-x-y}{\mathrm{Sr}}_{x}{\mathrm{Ce}}_{y}O_{2-\delta }N_{z}$$

$$\mathrm{NP}\left(\mathrm{Ba}\right):{\mathrm{Ti}}_{1-x-y}{\mathrm{Ba}}_{x}{\mathrm{Ce}}_{y}O_{2-\delta }N_{z}$$

$$\mathrm{NP}\left(\mathrm{Na}\right):{\mathrm{Ti}}_{1-x-y}{\mathrm{Na}}_{x}{\mathrm{Ce}}_{y}O_{2-\delta }N_{z}$$

$$\mathrm{NP}\left(\mathrm{Mg}\right):{\mathrm{Ti}}_{1-x-y}{\mathrm{Mg}}_{x}{\mathrm{Ce}}_{y}O_{2-\delta }N_{z}$$

$$\mathrm{NP}\left(\mathrm{Rb}\right):{\mathrm{Ti}}_{1-x-y}{\mathrm{Rb}}_{x}{\mathrm{Ce}}_{y}O_{2-\delta }N_{z}$$

### Synthesis of Single-Doped Anatase Phase

The method for synthesizing single-doped anatase phases with various elements, including strontium (Sr), cerium (Ce), and nitrogen (N), followed the same procedure outlined for synthesizing photocatalysts, using the exact quantities as described in the Synthesis of Photocatalysts section. However, only one dopant was incorporated into the anatase matrix for each synthesis.

### Photocatalytic Reaction

The photocatalytic reaction was conducted in a lab-made photocatalytic reactor (Figure 1). First, 0.0167 g L^–1^ of dye was prepared (in 60 mL of water) and stirred for 4 h. Before the addition of the photocatalyst and exposure to light, 3 mL of the solution was collected (first withdrawal). Next, 0.03 g of photocatalyst was added and left in the dark for 20 min (second withdrawal). The suspension was irradiated for 1 h using a 10 W LED installed on the walls of a box for 1 h, followed by exposure to a 300 W LED (9.55 mW cm^–2^ intensity; VPCRT6) located on the roof of the box for an additional hour (based on preliminary tests, the roof-mounted LED lamp was selected for subsequent experiments). Two withdrawals were made in the first hour, once every 30 min. A single withdrawal was performed after 60 min of LED lamp exposure. This strategy was implemented during testing across various conditions. Finally, UV–vis spectroscopy was performed to measure the photocatalytic degradation and collect all the kinetic data. The absolute intensity of the LED lamp and the light used in this experiment were calculated (Table S9). The results showed that the lamp intensity was even lower than that of the standard LED lamp used for photocatalytic degradation.^39^

**Figure 1:**
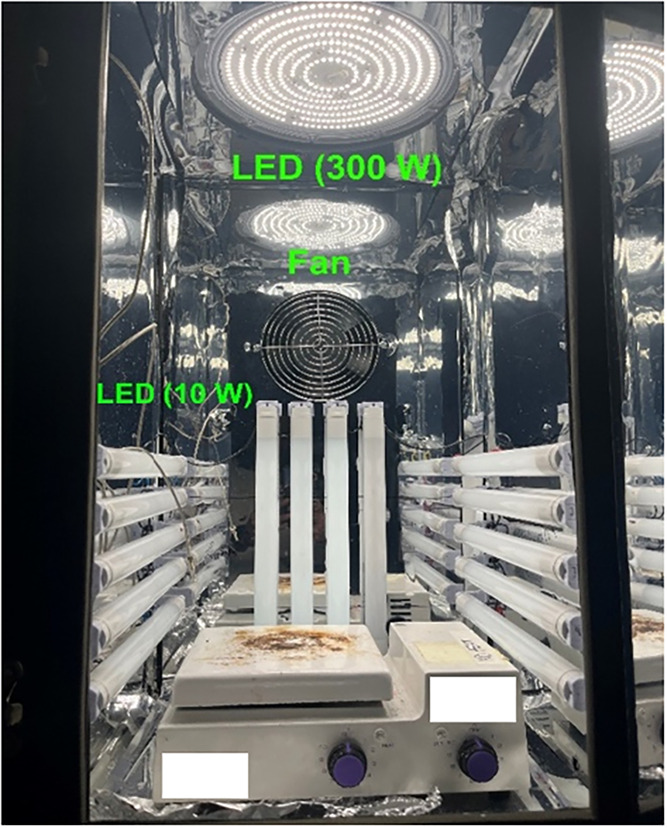
Lab-made photocatalytic box.

### Cyclic Voltammetry Measurement and Photocurrent Transition for Photocatalysts

A three-electrode system was used for cyclic voltammetry measurements, consisting of a reference electrode (3 M Ag/AgCl), a counter electrode (platinum wire), and a working electrode (glassy carbon electrode, diameter = 2 mm). The electrolyte solution consisted of chitosan dissolved in a 1% acetic acid solvent, which was then shaken for 3 days to form a highly viscous liquid. It was filtered to separate the undissolved particles. Then, 5 mM of iodine solution at 0.1 N was added to make the solution conductive, and it was stirred for an additional 20 min to obtain a homogeneous red solution. The solution was heated at 60 °C for 20 min to increase viscosity, then cooled and centrifuged to remove undissolved particles. Finally, 0.3 g of NP­(Sr) was added and stirred for 20 min. The particles were dispersed in the solution. Subsequently, N_2_ gas was purged for 15 min to saturate the surroundings. The scan rate was set at 0.01 V s^–1^, with two segments, a sensitivity of 1 × 10^–4^ A V^–1^, and the potential window was set from −1 to 1.2 V. For the photocurrent transition, at a potential of 0.8 V, a lamp, with the same intensity used for the photocatalytic degradation, was used to produce the photocurrent transition. The on and off durations varied between 0 and 275 s.

### Electrochemical Analysis

50 mg of the sample was added to the disposable SPE. Then, 2.5 mL of an ethanol: water mixture (4:1 v/v) was added. Both Mott–Schottky Electrochemical Impedance Spectroscopy (Mott–Schottky EIS) and potentiostatic EIS were carried out using the 1010E Interface (Gamry Instruments, Warminster, PA). The SPE electrodes were washed using deionized water and dried for 30 min. All measurements were performed at 25 ± 2 °C using 5 mM Na_2_SO_4_ as an electrolyte. Before both EIS measurements, the electrochemical system was stabilized at open-circuit potential for 10 min in dark conditions. Mott–Schottky EIS was performed at 500 Hz frequencies with a 5-mV amplitude.

Potentiostatic EIS was performed over the frequency range of 1 Hz to 100 kHz with an alternating current (AC) amplitude of 5 mV. The resulting impedance spectra were recorded as Nyquist plots, representing the imaginary component of the impedance (−*Z*_img_) as a function of the fundamental component (*Z*_real_). An equivalent of the Randle circuit was used to fit the EIS spectra, and it consists of a solution resistance (*R*_s_) in series with the combination of charge transfer resistance (*R*_ct_) and a constant phase element (CPE), which represents the nonideal capacitive behavior at the electrode/electrolyte interface.

### EPR Analysis

EPR studies were performed at room temperature in the X-band using a Bruker EMXplus. The EPR resonator was filled with sample suspensions ranging in volume from 10 to 15 μL. The experimental parameters were a microwave power of 0.6 mW, a microwave frequency of 9.87 GHz, a modulation amplitude of 0.04 Gs, and a frequency of 100 kHz. NP­(Sr) was suspended and exposed to optical irradiation using a 415 nm wavelength, 1 mJ optical parametric oscillator from Solar LS, and a 100 Hz repetition rate pulsed Nd:YAG LQ629 laser from the same source.

The EPR method was employed to detect the radical and its type, and Tiron (4,5-dihydroxy-1,3-benzene-disulfonic acid disodium salt, Sigma) was used as a probe. The Tiron concentration in the prepared solutions was 50–100 mM L^–1^. Superoxide was created when NP­(Sr) was exposed to visible light (1 mg per 60 μL of distilled water).

### Optimization of the Photocatalytic Reaction

We applied standard and statistical methods to obtain the optimization conditions. The standard procedure used to optimize the best conditions for photocatalyst performance typically employs three levels as effective parameters: temperature, dye concentration, catalyst dosage, and pH. In the statistical method, we aimed to identify the interaction between different parameters to determine the most crucial parameters and how these effective parameters interact to investigate their roles accurately. A multiple linear regression model was fit to the data.^40^ Four different variables (pH, dye concentration, catalyst dosage, and temperature), each at three levels, set 81 conditions for the study. We tested all of the conditions mentioned, and each was set to 2 h (Table S1).

## Results and Discussion

### Structural Characterization

XRD analysis identified the crystallite structure, as shown in Figures 2a and S1a. The results indicate that all photocatalysts were present in the anatase phase (JCPDS: 04-016-2837). All peaks shifted to higher angles relative to pristine anatase, which has a central peak at 25.3°. The (101) peak shifted from 25.3° (pristine anatase) to 29.3° for NP­(Sr), corresponding to a lattice contraction of approximately 13%. The estimated interplanar spacing of 0.3 nm corresponds to the (101) plane of anatase TiO_2_, confirming that the anatase crystal structure is retained despite multielement doping (Figure 3g). This peak shift demonstrates the influence of dopants on the TiO_2_ lattice, producing measurable contraction and compressive strain. The crystallite size of the photocatalysts was obtained by the Scherrer equation (eq 1):

1 $$\tau =\frac{K\lambda }{\beta \;\,\cos\theta }$$

Where β is the full width at half-maximum of the peak in radians, *K* is the shape factor, typically taken as 0.89, λ is the X-ray wavelength (in nanometers), and θ is the Bragg angle. The result obtained from the Scherrer equation showed that the crystallite size of the photocatalysts is mostly below 10 nm, with the smallest size belonging to the strontium-doped photocatalyst (Table 1).

**Figure 2:**
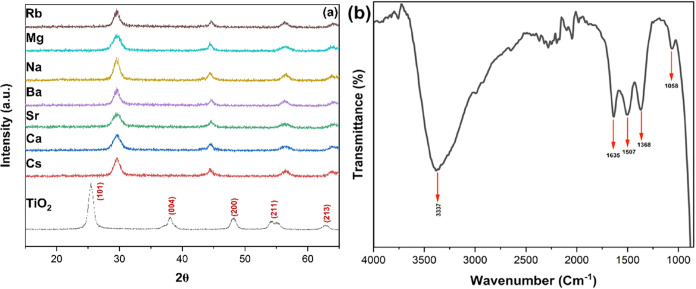
(a) XRD and (b) FT-IR spectra of the photocatalysts of nanoparticles (NPs).

**Figure 3:**
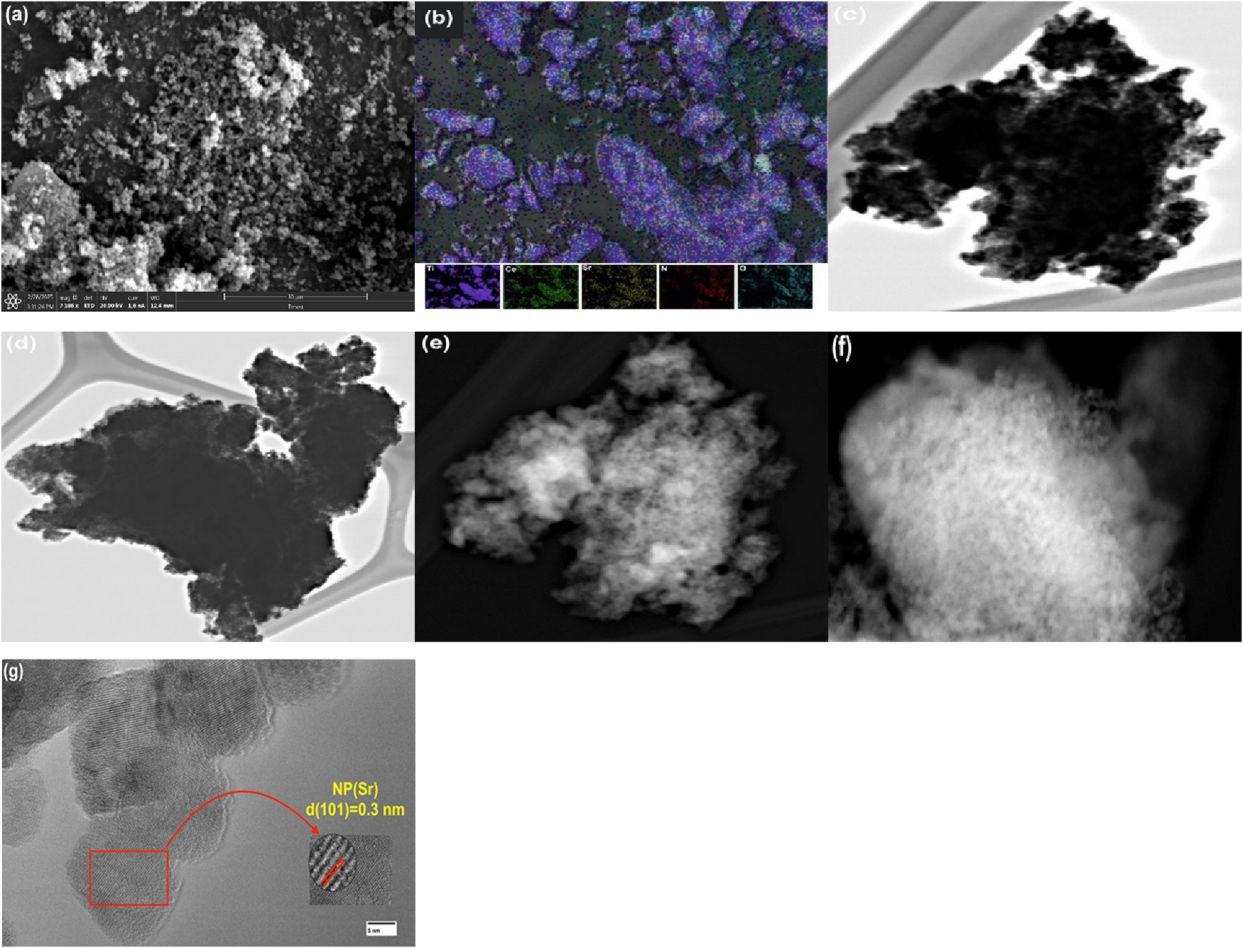
(a) FESEM, (b) EDS, (c, d) BF-STEM, (e, f) HAADF-STEM, and (g) HRTEM images of NP­(Sr).

**Table 1:** Structural and Textural Properties of Photocatalysts Determined by XRD, BET, and BJH Analyses

	NP(Cs)	NP(Ca)	NP(Sr)	NP(Ba)	NP(Na)	NP(Mg)	NP(Rb)
crystallinity (%)	99.37	98.79	98.79	98.55	99.05	99.45	98.95
crystallite size (nm)	7.86	5.35	5.17	8.64	7.53	5.7	61.7
BET Analysis							
*V*_m_ (cm^3^ g^–1^)	23.807	27.921	24.241	20.563	27.121	31.217	17.345
*a*_s,BET_ (m^2^ g^–1^)	103.62	101.52	105.51	89.502	98.04	104.87	75.493
total pore volume (cm^3^ g^–1^)	0.20447	0.2077	0.2152	0.2029	0.2103	0.2011	0.215
mean pore diameter (nm)	9.468	12.433	8.16	14.429	12.209	15.045	10.862
BJH Analysis							
*V*_p_ (cm^3^ g^–1^)	0.2705	0.3991	0.2319	0.3337	0.3749	0.538	0.2353
*r*_p,peak_ (area; nm)	3.09	4.61	2.1	4.03	4.61	4.03	4.03
*a*_p_ (m^2^ g^–1^)	109.46	109	154.9	126.56	154.55	142.28	131.2

Further discussion on the reasons behind this shrinkage in crystallite size can be found in the following sections. The FT-IR spectrum was obtained to investigate the midrange of the IR region (4000–500 cm^–1^) (Figures 2b and S1b). The spectrum of the photocatalysts exhibits a broad peak at 3337 cm^–1^, corresponding to the stretching of water molecules adsorbed on the photocatalyst surface. An absorption band at approximately 1635 cm^–1^ (δ­(OH)) indicates that there is hydration present on the surface of the sample. The peaks observed at 1058 cm^–1^ (low-frequency region) and 1368 cm^–1^ indicate that N–O-type species are incorporated within the anatase network rather than merely being adsorbed on the surface. These features indicate interstitial nitrogen incorporation, where N occupies positions between Ti and O atoms within the lattice.^41^ XPS analysis also confirmed this behavior, in which the nitrogen peak did not appear on the surface of NP­(Sr). The sharp peak at 1507 cm^–1^ belongs to N-related vibrations in doped structures.^42^

The morphology of the photocatalysts was examined using FESEM and STEM, accompanied by bright-field and HAADF imaging (Figures 3 and S2). According to the analysis, the photocatalysts display an amorphous, sheet-like surface morphology (Figure 3a). This surface structure is a result of the addition of ethylenediamine during the synthesis process. Ethylenediamine coordinates metal ions and selectively binds to specific crystal facets, thereby modulating crystal growth and leading to two-dimensional (2D) nanosheet morphologies.^43,44^

This strategy aimed to improve the photocatalytic capability of the surface, particularly with regard to its sheet-shaped form. In addition, EDS analysis was complemented by FESEM analysis to identify the elements present in NP­(Sr) (Figures 3b and S2). The standard deviation was measured to obtain the size of the NP­(Sr) through FESEM (Table S2). On the basis of this calculation, the particle size is 22.5 nm. On the basis of the EDS analysis and mapping, the corresponding elements belonging to NP­(Sr) are observable.

The BF-STEM image shows that NP­(Sr) clusters agglomerate and display irregular shapes due to the presence of multiple dopants (Figure 3c,d). The HAADF-STEM image illustrates their morphology and elemental distribution (Figure 3e,f), with the contrast reflecting uniform multidopant dispersion in the anatase phase. Bright regions indicate areas of higher atomic number density, confirming the successful incorporation of the dopants. The agglomerated morphology and BF-STEM observations provide profound insights into the structural uniformity and potential active sites for the photocatalytic applications. The interplanar spacing was measured from HRTEM images using ImageJ software (NIH) with calibration against known lattice parameters (Figure 3g). The *d*-space measured is equal to 0.3, which shows a contraction from 0.35 nm, corresponding to the (101) face of anatase TiO_2_. This lattice distance matches the X-ray diffraction (XRD) spectra (Figure 1a).

An XPS survey scan was performed to examine the surface properties of NP­(Sr) (Figure 4a). The doublet associated with Ti’s 2p fits quite well with the TiO_2_ (458.41 eV for the Ti 2p_3/2_), which authenticates the presence of Ti^4+^ (Figure 4b).^45^ Figure 4c shows the high-resolution spectra of Ce 3d. The position of the lowest binding energy Ce 3d feature is at 881.36 eV, which, along with the line shapes of the Ce 3d spectra, indicates good agreement with the Ce^3+^ state.^46^ The presence of Ce^3+^ facilitates electron transfer and helps enhance visible-light absorption through the electronic structure of the anatase phase.^47,48^ For strontium, the Sr 3d doublet at 133.3 eV (Figure 4d) corresponds to Sr^2+^ species, consistent with SrCO_3_-like environments. The larger ionic radius of Sr^2+^ (1.18 Å) relative to Ti^4+^ (0.61 Å) introduces significant lattice strain, stabilizing the anatase phase and generating oxygen vacancies.

**Figure 4:**
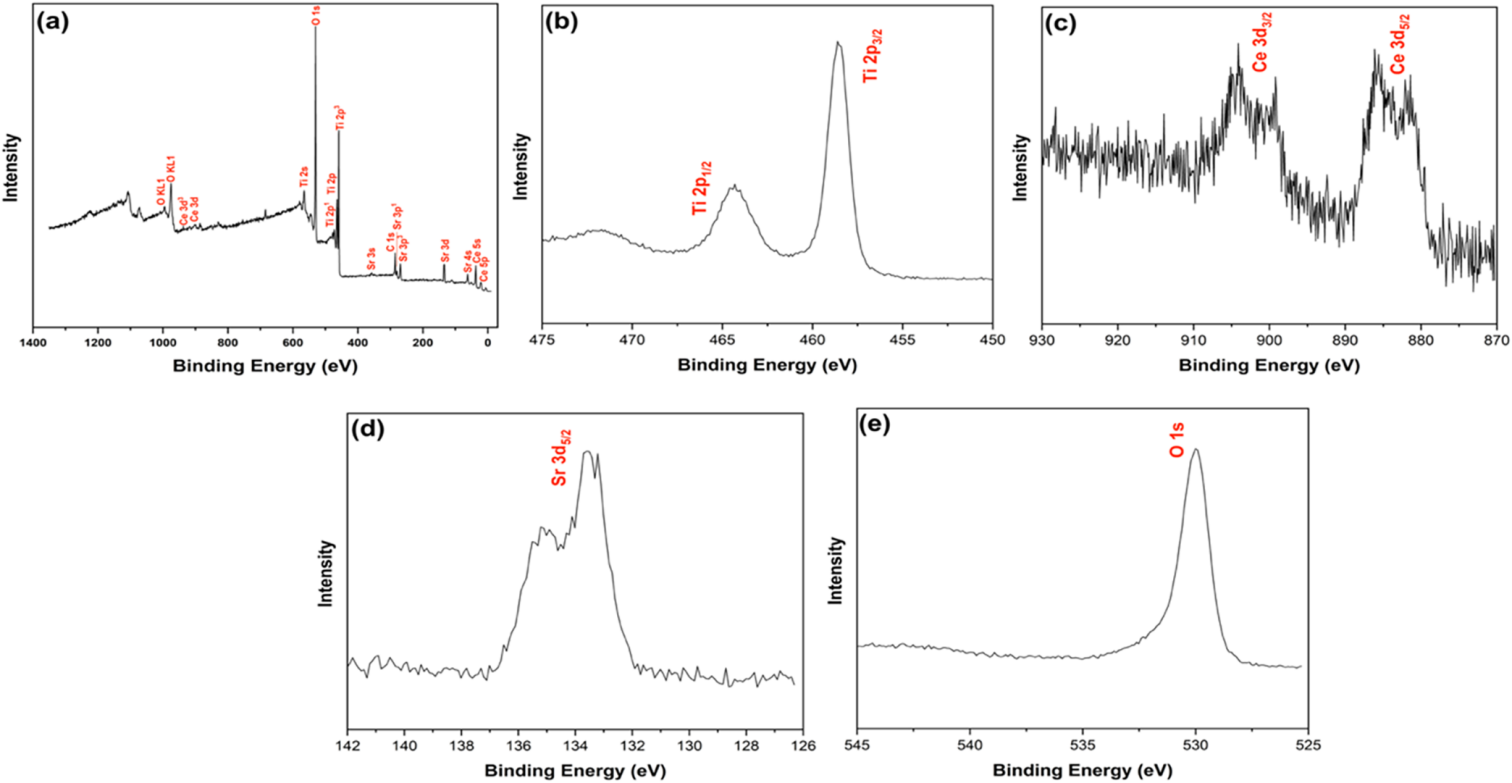
(a) XPS spectrum of NP­(Sr), (b) 2p Ti core level, (c) 3d Ce core level, (d) 3d Sr core level, and (e) 1s O core level.

On the other hand, the O 1s spectrum presents a single peak at 5329.9 eV, which is attributed to the lattice oxygen in titanium oxide (Figure 4e). A slight shoulder is observed at higher binding energy, which may result from a combination of SrCO_3_ and organic contaminants. No N 1s signal was detected at the surface, so it appears that although nitrogen is in the bulk of NP­(Sr) (Table 2), it did not appear on the surface of NP­(Sr).^49–53^ It confirms that nitrogen was distributed mainly within the core or beneath the surface layer, which was not detectable by XPS, as it could only observe the element on the surface at a maximum depth of 10 nm. Although nitrogen has not appeared on the surface of NP­(Sr), it helps reduce the energy band gap, leading to the absorption of higher visible wavelengths (Figure 5b).

**Figure 5:**
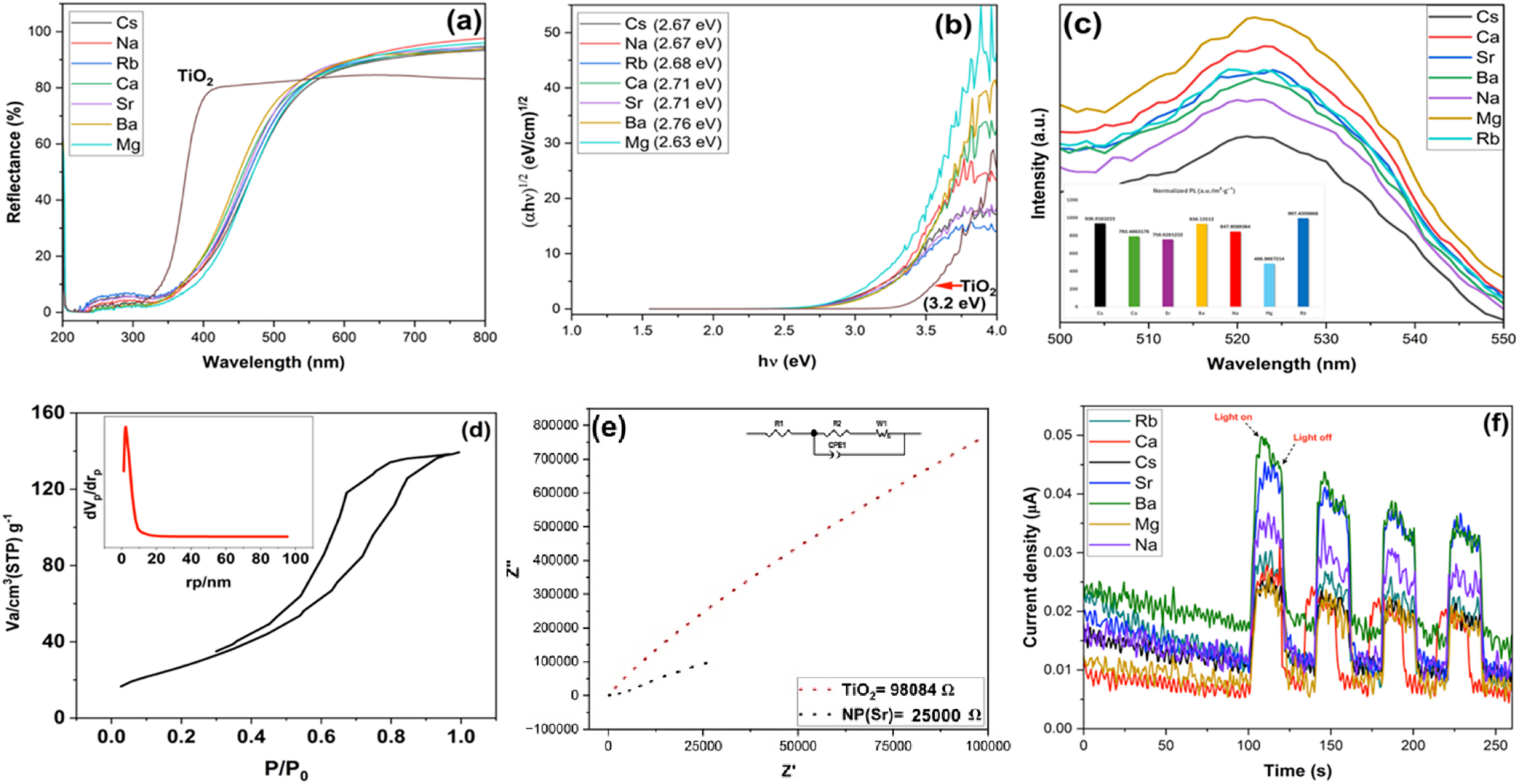
(a) Reflectance analysis, (b) energy band gap, and (c) photoluminescence emission spectra (λ_ex_ = 380 nm, refer to its UV–vis spectrum) of the doped anatase phase with Ce, N, and alkali or alkaline-earth, (d) surface and porosity study and (inset) the pore diameter, (e) charge-transfer dynamics of the samples measured using electrochemical impedance spectroscopy (EIS); inset shows the equivalent Randles circuit used for fitting, and (f) transient photocurrent analysis of NP­(Sr).

**Table 2:** Chemical Composition of NP­(Sr)

compound	Ti, %	Ce, %	Sr, %	N, %	O, %
Ti*_a_* Sr*_b_* Ce*_c_* N*_d_* O*_e_*	28.5	5	10	4.5	52

Elemental analysis was conducted through CHN and ICP analyses to determine the amounts of the elements. On the basis of these analyses, all the corresponding elements exist in NP­(Sr) (Table 2 and Figure S2).

### Optical and Porosity Properties

Diffuse reflectance spectroscopy (DRS) was utilized to assess the optical activity of the photocatalysts within the 200–800 nm wavelength range (Figure 5a). Based on the result, all the photocatalysts could absorb visible light between 425 and 495 nm. In addition, the energy band gap of the photocatalysts was calculated through the Tauc plot (eq 2).

2 $$\alpha ={\alpha_{0}\left(h\nu -E_{g}\right)}^{n}/h\nu$$

Where α is the absorption coefficient, *h*ν shows the photon energy, α_0_ and *h* are constants, *E*_g_ denotes the optical band gap of the material, and n ranges between 0.5 and 3 depending on the electronic transition. Plotting (α*h*ν)^1/2^ vs *h*ν yields the energy band gap of the photocatalysts. Based on the “*n*”, which equals 0.5, the band gap is determined by the *h*ν value at the tangent–*x*-axis intersection.

Tauc plot analysis shows that the photocatalysts exhibit bandgaps between 2.5 and 2.9 eV, confirming visible‑light absorption. (Figure 5b). This is crucial in determining how each of these dopants affects the reduction of the energy band gap. Therefore, it was tested by introducing a single dopant into the anatase matrix, and the results can be seen in the “Role of Effective Parameters” section.

The recombination rate of the photocatalysts was measured by photoluminescence analysis (PL) (Figure 5c). On the basis of the results, NP­(Cs) has the lowest intensity compared to other photocatalysts, which confirms that the rate of electron recombination from the conduction band to the valence band is the least, allowing electrons to have more time to react in the conduction band. On the other hand, NP­(Mg) exhibits the fastest recombination due to its highest intensity in PL compared with the others. NP­(Sr) showed a moderate rate on the basis of its intensity.

The surface area, mean pore diameter, and total pore volume have been measured using a typical N_2_ adsorption–desorption isotherm of the BET method and the BJH method (Figure 5d, Table 1). The porosity type was obtained by measuring the pore volume in the *p*/*p*_0_ range of 0–0.99 (Figures 5d and S3). Based on the pseudo-V isotherm obtained from the BET method, a strong and weak adsorption–desorption interaction can be seen in the isotherm. The initial region of the isotherm (0.1–0.4) suggests a strong adsorption interaction, which occurs at low relative pressure. The linear region indicates moderate adsorption in the medium relative pressure (0.4–0.9). The hysteresis loop resembles an H2-type loop, indicating that the pore structure most likely resembles ink-bottle shapes. The narrow necks of the pores cause a delay in desorption, resulting in a steeper slope of the desorption branch. The nanophotocatalysts’ surface area is between 75.49 m^2^ g^–1^ (NP­(Rb)) and 105.51 m^2^ g^–1^ (NP­(Sr)) (Table 3 and Figure S3; inserted figure). The mean pore diameter of the samples confirmed that the nanocrystals are mesopores (2–50 nm diameter) (Table 3, Figure 5d; inserted and Figure S3 inserted). NP­(Sr) shows the highest total pore volume (0.2152 cm^3^ g^–1^) and the smallest mean pore diameter (8.16 nm) compared to other samples, which was also confirmed by BJH analysis (Figure S3). It leads to high reactivity, improved light absorption, enhanced charge separation, better stability, and more efficient pollutant degradation.^54–56^

**Table 3:** Degradation of Methylene Blue by Different Photocatalysts under Visible Light

	NP(Cs)	NP(Ca)	NP(Sr)	NP(Ba)	NP(Na)	NP(Mg)	NP(Rb)
degradation (%)	74.86	56.42	around 100	49.62	65.47	72.23	81.35
*k*_app_ (min^–1^)	0.014×	0.0094×	0.0205×	0.0071×	0.0104×	0.0126×	0.0168×
*R* ^2^	0.9916	0.9469	0.8185	0.9684	0.9317	0.985	0.9725

average of three run tests				
	first run	second run	third run	average
degradation (%)	100	93.7	97.9	97.2
*k*_app_ (min^–1^)	0.0205×	0.05784×	0.0736×	
*R* ^2^	0.8185	0.85	0.89	

The integrated photoluminescence (PL) was normalized through the BET surface area to investigate the recombination of electrons and holes (Figure 4c, inserted). This is an appropriate method to remove the effect of the surface area and provides a purer materials’ electronic behavior. This is because PL result is not just influenced by the recombination, but other parameters are also affect the results, including particle size, surface texture, and how much light the material can absorb. So, by normalizing the data, comparisons focus on the materials’ intrinsic properties rather than physical variations. Using this approach, the PL spectra were integrated and adjusted based on the BET surface area to allow meaningful, quantitative comparisons. The results showed the following trend in normalized PL intensity: NP­(Mg) (486.97 au/(m^2^·g^–1^)) < NP­(Sr) (758.63 au/(m^2^·g^–1^)) < NP­(Ca) (793.49 au/(m^2^·g^–1^)) < NP­(Na) (847.96 au/(m^2^·g^–1^)) < NP­(Ba) (934.13 au/(m^2^·g^–1^)) < NP­(Cs) (936.92 au/(m^2^·g^–1^)) < NP­(Rb) (997.44 au/(m^2^·g^–1^)). This pattern confirms that the Mg- and Sr-doped samples have the lowest electron–hole recombination per unit of surface area.

Surprisingly, although NP­(Mg) has the lowest integrated PL intensity, its emission band is sharper and narrower, which naturally reduces the total PL area. In the case of NP­(Sr), low recombination is mainly due to strong electronic interactions between the Sr^2+^ ions and the TiO_2_ structure. Ti^4+^ is partially replaced with Sr^2+^ and creates shallow defects near the conduction band. These defects trap electrons, extending their lifetime and reducing the time for recombination of electrons. In addition, doping with Sr leads to moderate distortion in the crystal lattice and increases oxygen vacancies. Finally, these factors improve the transfer of charges to the dye molecules on the surface. As a result, NP­(Sr) provides a good balance between charge separation and surface reactivity, enhancing the photocatalytic performance under visible light.

Charge-transfer dynamics of the samples were studied using electrochemical impedance spectroscopy (EIS) (Figure 5e). The charge-transfer resistance (*R*_ct_) was extracted from the Nyquist plots by fitting the data with an equivalent circuit model. The fitted results revealed that TiO_2_ exhibited an *R*_ct_ of around 100,000 Ω, while it is 25,000 for NP­(Sr), which is much lower, illustrating a substantial enhancement in interfacial charge transfer after doping. The reduction in *R*_ct_ is attributed to the introduction of Sr, N, and Ce dopants into the TiO_2_ lattice, which provides multiple electronic states within the band gap and enhances electronic conductivity. Among the dopants, Sr^2+^ plays the primary role for lattice charge balance, substituting Ti^4+^ and generating oxygen vacancies that facilitate charge migration. Nitrogen introduces shallow acceptor levels that narrow the band gap, thereby improving visible-light absorption. Ce ions can reversibly transition between Ce^3+^ and Ce^4+^ states, functioning as dynamic electron mediators that promote charge transfer and reduce carrier recombination. The synergistic interaction among these dopants stabilizes defect states, reduces the overall charge-transfer resistance, and enhances the interfacial electronic conductivity of TiO_2_.^57,58^ The Randles equivalent circuit was used to fit the result of the impedance spectra (Inserted in Figure 5e). The Nyquist response was fitted with a Randles equivalent circuit to determine the values of the constant phase element (CPE1), the diffusional resistance element (the Warburg impedance, *W*_1_), the ionic solution’s Ohmic (*R*_ohm_, here *R*_1_) resistance, and the electrode’s charge transfer (*R*_CT_) resistance. Here it shows as *R*_2_.

The transient photocurrent of the samples was measured to study the behavior of the charge carrier (Figure 5f). The higher photocurrent density indicates that a large number of charge carriers can be generated under light exposure. The result confirmed a significantly higher photocurrent density of NP­(Sr) and NP­(Rb) compared to other samples. After passing time, NP­(Sr) showed even a higher photocurrent density than NP­(Rb). This high photocurrent density exhibited better photocatalytic activity, as it generated more electron–hole pairs, which play a leading role in the photocatalytic reaction. Additionally, it demonstrates higher material efficiency by showing how a material can convert absorbed photons into charge carriers. Besides, the taller peak obtained by NP­(Sr) suggests a more efficient initial separation of the electron–hole pair before recombination. The time rate from the light on and off is longer for NP­(Sr), which shows a lower electron–hole recombination rate, which is crucial for completing the reaction. Although the absolute photocurrent values are low, this is expected under such low-intensity LED irradiation (9.55 mW cm^–2^) and with a powder-coated electrode configuration; the reproducible light on/off response confirms the validity of the photo response measurement.

## Photocatalytic Study

The obtained photocatalysts were tested under the same conditions for the initial comparison (as discussed in Notation and Dopant Incorporation). Then, the most efficient photocatalyst was applied in various situations to determine the optimal conditions. On the basis of the result, NP­(Sr) showed a distinguished ability to reduce the concentration of methylene blue (Figure 6). pH, dye concentration, catalyst dosage, and temperature are the key factors that significantly influence the photocatalytic degradation of the textile dyes.

**Figure 6:**
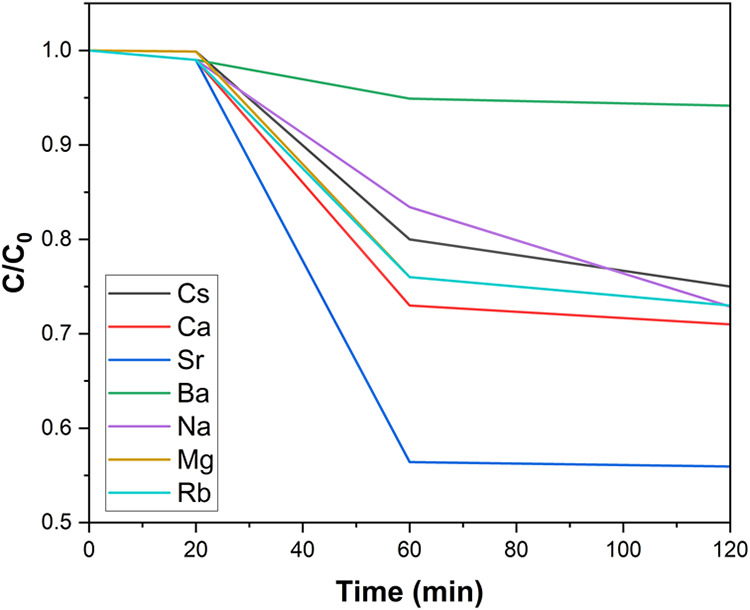
Photocatalytic efficiency of the obtained photocatalysts under visible light.

### Effect of pH

Photocatalytic degradation of methylene blue was studied at acidic, neutral, and basic pH levels (Figure S6). HCl and NaOH created acidic and basic pH conditions of 2 and 11, respectively. On the basis of the result, basic and neutral environments were more effective in reducing methylene blue than acidic ones. Under acidic conditions, 2% of methylene blue was degraded after 120 min, while it was 42 and 82% under neutral and basic conditions, respectively. Therefore, the basic condition was chosen for the following condition.

### Effect of Dye Concentration

In this step, three different concentrations of 0.0167, 0.0083, and 0.0333 g L^–1^ were compared under basic conditions with 0.03 g of NP­(Sr) (Figure S7). The first two concentrations of 0.0167 and 0.0083 g L^–1^ were reduced by almost the same amount, with minimal differences (82 and 85%, respectively), which we can assume as the error. On the other hand, it was 30% for the concentration of 0.0333 g L^–1^. Therefore, 0.0167 g L^–1^ was selected for subsequent experiments.

### Effect of the Catalyst Dosage

This step compares catalyst dosages of 0.001, 0.0075, 0.015, and 0.03 g (Figure S8). During this stage, the dosage of NP­(Sr) systematically decreased to achieve a targeted outcome. According to the results, the highest reduction was obtained with dosages of 0.0075 and 0.015 g (97%), an 83% reduction was achieved with 0.03 g, and finally, the minor reduction was obtained with 0.001 g, at around 30%. Therefore, 0.0075 g was chosen for the next step.

### Effect of Temperature

This step tested different temperatures, including room temperature (30 °C), 45 °C, and 60 °C (Figure S9). The result showed that increasing the temperature affected the result, and the concentration reduction was reduced by increasing the temperature. In the range between 30 and 45 °C, the concentration of dye decreased equally. However, increasing the temperature to 60 °C results in a lower reduction in the concentration of methylene blue.

According to the result, the highest reduction was obtained at 30 and 45 °C (100%), while it was around 75% when the temperature was increased to 60 °C.

### Photocatalytic Activity Comparison

After evaluating the best condition (pH: basic conditions, concentration of dye: 0.0167 g L^–1^, dosage of catalyst: 0.0075 g, and temperature: 30 °C), the final comparison between different oxide nanocrystals was carried out (Figures 7 and S4). The result showed that all the synthesized photocatalysts can remove methylene blue from water (Figure S4, Table 3). However, NP­(Sr) can completely remove methylene blue from water (Figure 7a,b). The photocomposition efficiency indicated that NP­(Sr) could reduce the concentration of methylene blue to almost zero (Figure 7c). Following this, NP­(Ba), NP­(Cs), and NP­(Mg) demonstrated a strong ability to remove 80% of methylene blue from the water. All photocatalytic degradation experiments were performed in triplicate, and the data are presented as mean values with corresponding error bars representing the standard deviation (Figure 7e,f and Table 3).

**Figure 7:**
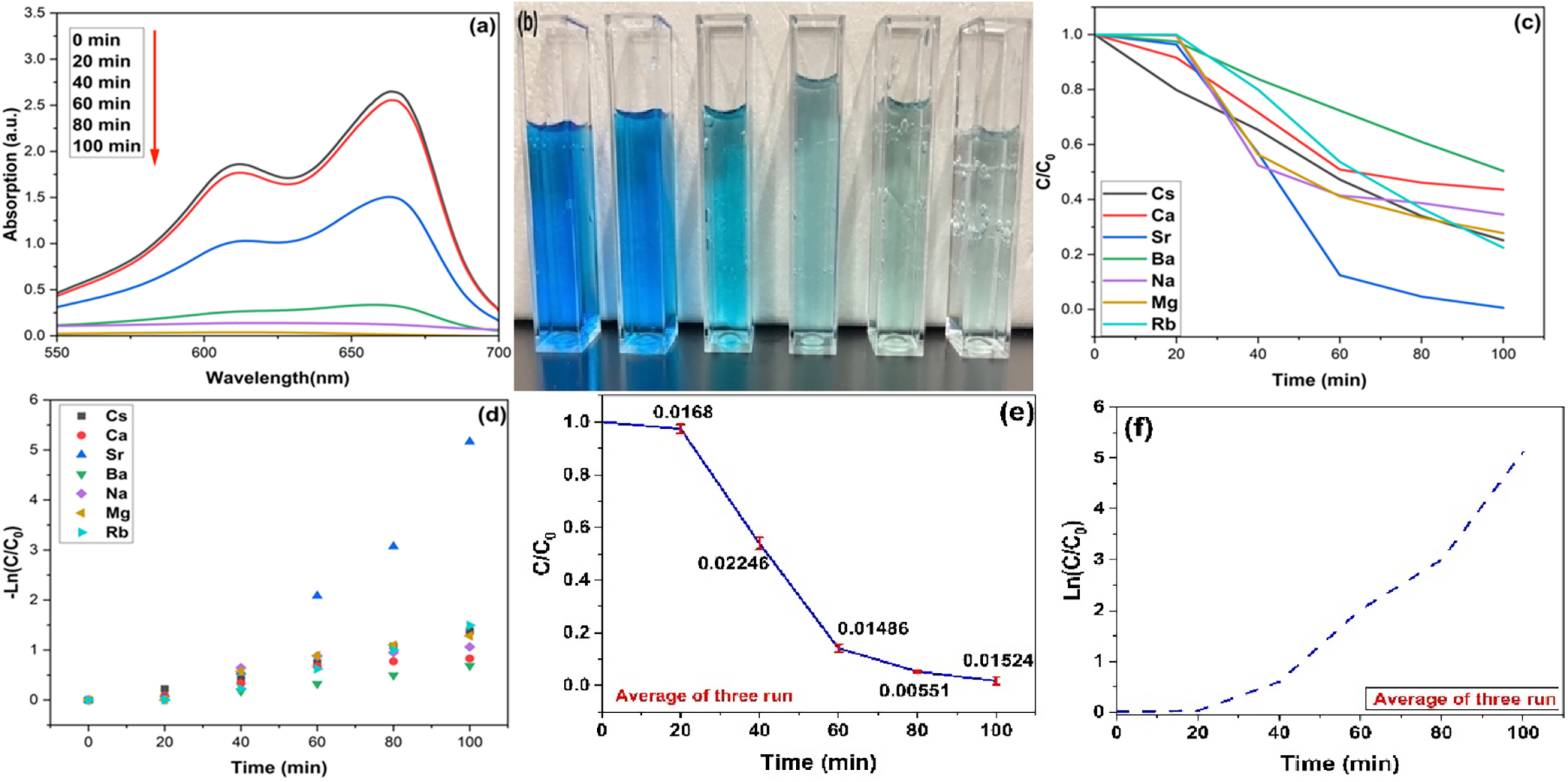
(a) Photocatalytic degradation of methylene blue by NP­(Sr), (b) reducing the concentration step by step, (c) photodecomposition efficiency of different nanophotocatalysts, (d) photocatalytic degradation kinetics of the nanophotocatalysts under visible light, (e) the average of photodecomposition efficiency of NP­(Sr) after three runs, and (f) the average of photocatalytic degradation kinetics of NP­(Sr) under visible light after three runs. Error bars represent the standard deviation of three independent experiments (*n* = 3).

### Kinetic Study

The Langmuir–Hinshelwood model is a standard method for studying the kinetics of photocatalytic degradation.^59^ The model was first used for gas–solid systems. However, the model has been extended to be used for solid–liquid systems as well (eq 3):

3 $$\ln\left(C_{0}/C\right)=k_{\mathrm{app}}\times t$$

In this equation, *C*_0_ is the initial concentration of methylene blue after the adsorptive equilibrium (g L^–1^), *C* is the measured concentration of methylene blue (g L^–1^) at time *t*, and *k*_app_ is the pseudo-first-order rate constant (min^–1^). *k*_app_ can be obtained from the slope when a plot of ln­(*C*/*C*_0_) is made on the *y*-axis against time on the *x*-axis (Figure 7d). The kinetic study was conducted for all the mentioned steps, and the results are presented in Table S4 (Figure S5).

The total organic carbon (TOC) was measured to study the amount of carbon and see if it was just a discoloration or mineralization (Table S3). The result confirmed the initial TOC concentration of the 100% MB solution is 2.573 mg L^–1^. The TOC analysis was done for different samples obtained from different photocatalytic degradation times, and it shows that TOC decreased to 0.281 mg L^–1^, and based on the mineralization efficiency, it is approximately 10.9 mg L^–1^ (89%). The pure water was also considered as the blank sample, and the TOC of that was measured as 0.183 mg L^–1^, which indicates the residual organic carbon after degradation is very close to the pure water. The result confirmed that NP­(Sr) achieves effective mineralization rather than simple decolorization.

The results showed that NP­(Sr) had a higher *k*_app_ value compared to other doped nanocrystals, indicating that strontium doping significantly enhanced the photocatalytic activity.

### Photocatalytic Activity of Different Dyes with NP­(Sr)

In addition to methylene blue (a basic dye), this study investigated other dyes, including bromophenol blue (an acid dye), methyl orange (an azo dye), and reactive dyes, which were degraded photocatalytically by NP­(Sr). Using NP­(Sr) with the exact amounts of catalysts and dye concentrations at different pH levels demonstrated that the photocatalyst can effectively remove dyes from various categories.

Bromophenol blue, as an anionic triphenylmethane dye, demonstrates a degradation behavior opposite to methylene blue, with a remarkable degradation (90%), in the acidic environment (*k*_app_ = 0.0239 min^–1^, *R* ^2^ = 0.90) (Figure S10, Table S4). Based on the ζ potential of the surface (+38.3 mV), it is because of the electrostatic attraction with the anionic sulfonate groups of bromophenol blue, which facilitates direct contact between the dye and photogenerated reactive species. On the other hand, the degradation in the basic environment is low, around 15%, despite remarkable first-order kinetics (*R* ^2^ = 0.97), showing that although the degradation mechanism remains constant, the high electrostatic repulsion between bromophenol blue and negatively charged catalyst surface does not allow proper adsorption and degradation. The *R* ^2^ in the basic environment is high, which is because of the thermodynamic (adsorption) limitations rather than the mechanistic complexity.

In general, the degradation of methyl orange is less, especially in the basic and neutral conditions, around 15–30%, and it is better in the acidic environment, with 60% degradation and remarkably low first-order correlation coefficients (*R* ^2^ = 0.56–0.66), which is related to its azo bond (–N=N–) structure (Figure S11, Table S5). In general, the degradation of methyl orange, as an azo dye, started from cleavage of this bond, and it is followed by oxidation of aromatic intermediate, which has resulted in a complex kinetic that deviates from first-order behavior. In the same condition, methylene blue shows more degradation (*R* ^2^ = 0.97). Methylene blue, as a cationic thiazine dye, is illustrated strongly and in the best condition (basic environment). Under optimized conditions, the degradation is around 100%, with a *k*_app_ of 0.0205 min^–1^ and *R* ^2^ equal to 0.82. This remarkable degradation is because of the attraction between the positive charge of the dye and the negatively charged NP­(Sr) surface (ζ potential = −15.2 mV at neutral, −6.63 mV at basic pH). The enhancement in the adsorption leads to facilitating efficient electron transfer from NP­(Sr) to the dye, promoting superoxide-mediated degradation. On the other hand, in the acidic conditions, the result in degradation is very low (2%) because of the repulsion between the cationic dye and the positive charge catalyst surface (ζ potential = +38.3 mV). The significant first-order correlation, with *R* ^2^ = 0.82, under the basic environment, confirms a single dominant degradation pathway, which matches the proposed superoxide mechanism.

In the case of reactive red dye 120, which is an anionic dye with multiple sulfonate groups and reactive chlorotriazine moieties, the highest degradation is obtained in the acidic condition (100%, *k*_app_ = 0.0427 min^–1^, *R* ^2^ = 0.96) (Figure S12, Table S6). The significant degradation is because of the strong electrostatic attraction between the highly anionic dye (multiple −SO_3_^–^ groups), and the positively charged NP­(Sr) surface at low pH. In addition, the reactive chlorotriazine groups are nucleophilically attacked by superoxide radicals, which provides not a single but multiple pathways to accelerate removal. The excellent first-order correlation (*R* ^2^ = 0.96) confirms that although the structure of the red reactive dye 120 is complex, the degradation is possible through optimized conditions. The same scenario is also applicable for low degradation (10%) in the basic environment with a rate constant 2 orders of magnitude lower (*k*_app_ = 0.0006 min^–1^), which is electrostatic repulsion.

## Mathematical Study

### Preliminary Analysis

We fit the following regression model for the initial data set:

$$y=\beta_{0}+\beta_{1}\left(pH\right)+\beta_{2}+\beta_{2}\left(dye\;concentration\right)+\beta_{3}\left(catalyst\;dosage\right)+\beta_{4}\left(temperature\right)+\epsilon$$

Here, the response variable *y* is taken as the percentage of reduction. The regression coefficients are β_0_, β_1_, β_2_, β_3_, and β_4_, and ϵ represents the random error. Following the standard regression setup, the errors are assumed to be identically and independently distributed with zero mean and constant variance. The regression output is given below (Table 4):

**Table 4:** Coefficients of Different Parameters^a^

	estimate	St. error	*t*-value	*p*-value Pr(>|*t*|)
(intercept)	126.8764	14.1292	8.980	3.25 × 10^–13^
pH	–6.8712	0.9647	–7.123	7.96 × 10^–10^
dye concentration	9403.4000	26,450.1117	0.356	0.723
catalyst dosage	–304.1410	849.9079	–0.358	0.722
temperature	–0.1396	0.2189	–0.638	0.526

a Residual standard error: 23.17 on 69 degrees of freedom; multiple *R*-squared: 0.4292, adjusted *R*-squared: 0.3961; *F*-statistic: 12.97 on 4 and 69 DF, *p*-value: 6.269 × 10^–08^.

This is not a good fit, with *R* ^2^ = 42.9% (less than 50%), and the pH environment is strongly significant (*p*-value is 7.96 × 10^–10^). Hence, we fit the following regression model separately for neutral, acidic, and basic environments.

$$y=\beta_{0}+\beta_{1}\left(\mathrm{dye}\right)+\beta_{2}\left(\mathrm{catalyst}\right)+\beta_{3}\left(\mathrm{temperature}\right)+\epsilon$$

Generally, the temperature is statistically significant (*p* values ranging between 0.05 and 0.10). We thoroughly investigated the effect of temperature in our final analysis. The following plot (plot 1) clearly shows that the basic environment (pH = 11) is the most effective (Tables S7–S9), so we focused our attention on the basic environment (Figure 8).

**Figure 8:**
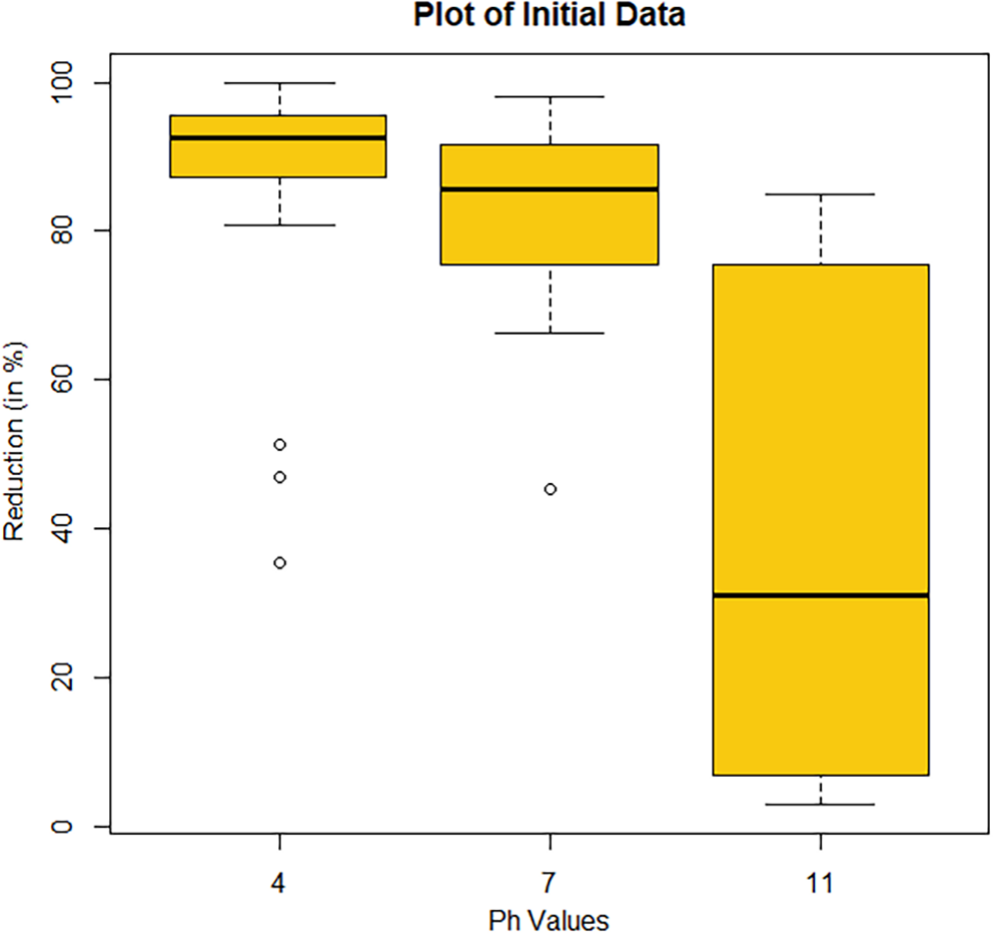
Result of the initial study.

As temperature also played a significant role, we repeated the study with a broader range of temperatures, from 30 to 70 °C, with an interval of 10 °C. The data are given below (Table S10):

We fit a regression model, which is a quadratic function of temperature, as follows:

$$y=\beta_{0}+\beta_{1}x+\beta_{2}x^{2}+\epsilon$$

The response variable *y* is the fourth power of concentration, based on the Box–Cox transformation. Here, *x* is the temperature. The regression output is given below (Table 5):

**Table 5:** Coefficients of Different Temperatures in the Basic Environment (pH > 7)^a^

	estimate	St. error	*t*-value	Pr(>|*t*|)
(intercept)	–79,811,437	31,970,442	–2.496	0.0281
temperature	8,917,579	1,352,237	6.595	2.56 × 10^–05^
(temperature)^2^	–108,562	13,429	–8.084	3.38 × 10^–06^

a Residual standard error: 8,703,000 on 12 degrees of freedom; multiple *R*-squared: 0.947, adjusted *R*-squared: 0.9381; *F*-statistic: 107.1 on 2 and 12 DF, *p*-value: 2.228 × 10^–08^.

The fitted model, along with the data, is plotted below. The regression output (*R* ^2^ = 94.7%, *p*-value = 2.22 × 10^–8^) and the plots clearly show that the quadratic polynomial model is a good fit, with both. β_1_ and β_2_ are strongly significant (*p*-values being approximately 0). Here we plot the time taken to reach the saturation point for different temperatures. The result confirmed that a temperature of 50 °C yields the highest efficiency in the shortest time (Figure 9).

**Figure 9:**
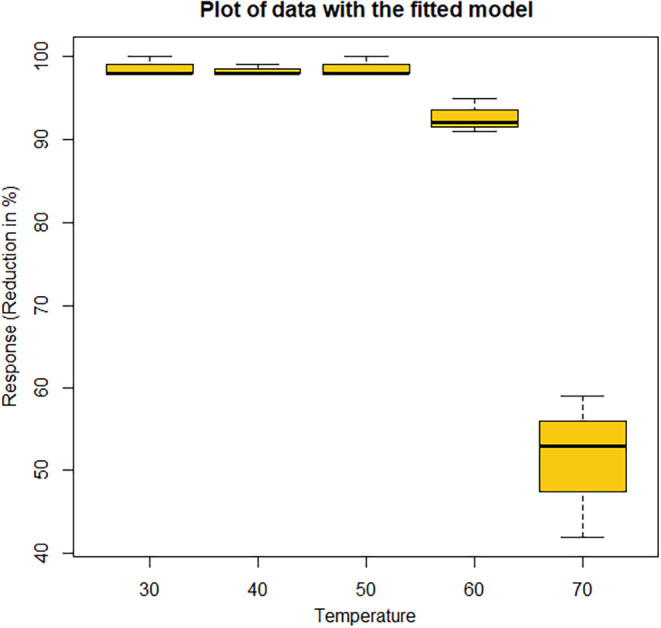
Degradation of dye at different temperatures.

## Role of Effective Parameters

Various effective parameters significantly impact the photocatalytic degradation of dyes in water, which can be divided into two main categories: photocatalyst- and environment-based.

### Photocatalyst-Based

These parameters relate to the photocatalyst and its physical and chemical properties. An investigation was conducted to assess the role of each element in key parameters, as shown in Table 6. Doping titanium oxide with one element at a time enables systematic isolation and investigation of each dopant’s effect on the titanium oxide matrix. This approach provides an in-depth analysis of the electronic structure, optical characteristics, and photocatalytic performance. The crystallite structure of the obtained compounds was investigated with XRD (Figure 10a). Based on the XRD pattern, all the samples have an anatase phase, so the influence of the dopants causes changes in the crystallite size (local lattice strain or distortion). The crystallite sizes of Ti*_a_* Ce*_b_* O*_c_* and Ti*_a_* N*_b_* O*_c_* are almost the same, with 11.41 and 11.69 nm, respectively (Table 6). It suggests that these elements expand the strain associated with the anatase phase due to interstitial incorporation, where Ce and N occupy lattice sites, leading to distortion.

**Figure 10:**
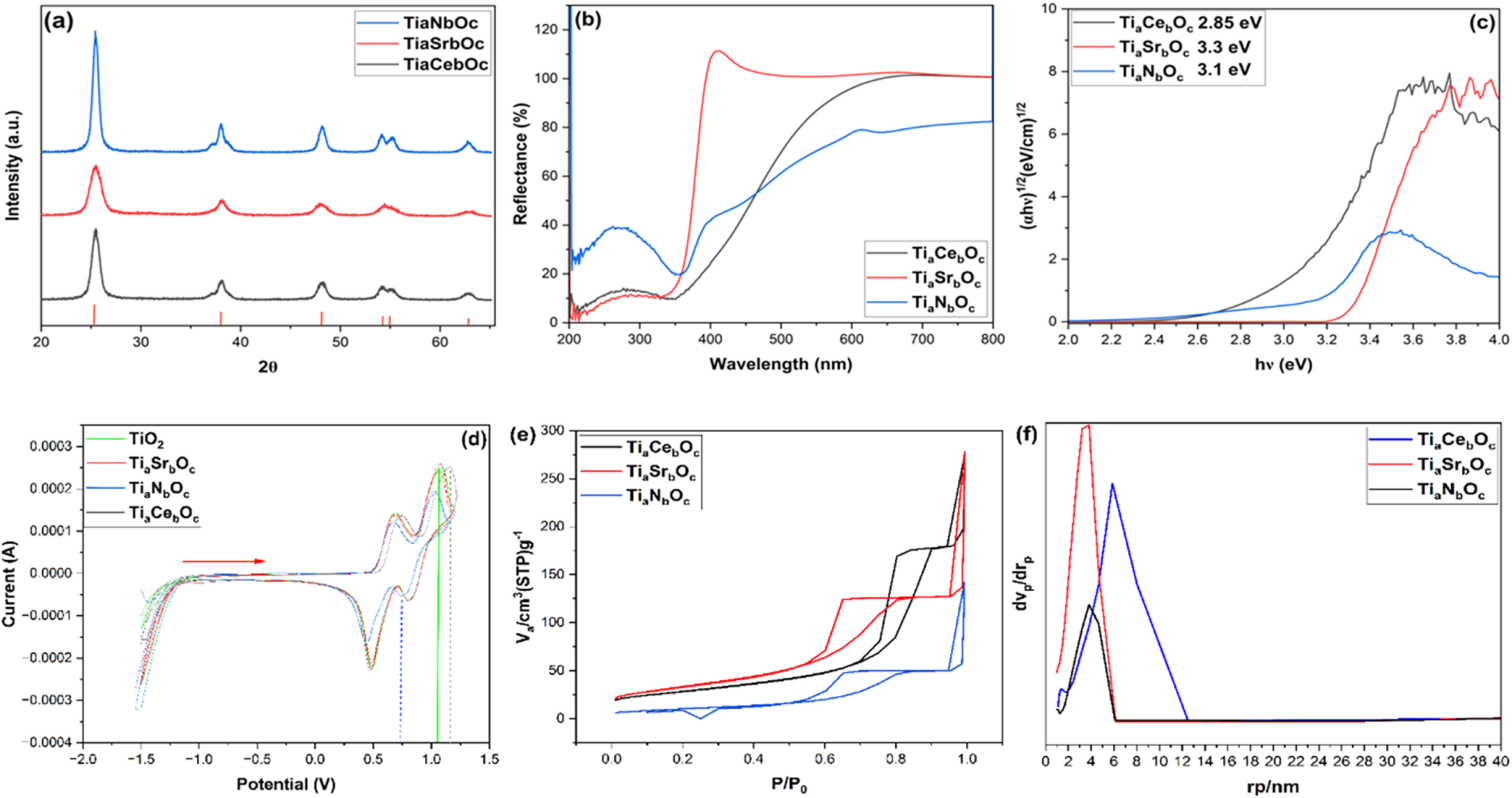
(a) XRD, (b) DRS, (c) energy band gap, (d) CV analysis, (e) surface and porosity (f), and the mean pore diameter study of different single dopants.

**Table 6:** Different Properties of Single Dopants

compound	crystallite size (nm)	BET surface area	mean pore diameter (nm)	total pore volume (cm^3^ g^–1^)	energy band gap (eV)
Ti*_a_* Ce*_b_* O*_c_*	11.41	100.72	6.38	0.42	2.85
Ti*_a_* Sr*_b_* O*_c_*	7.01	120.25	5.27	0.44	3.3
Ti*_a_* N*_b_* O*_c_*	11.69	38.42	7.03	0.21	3.1
TiO_2_	8.2				3.25

On the other hand, Ti*_a_* Sr*_b_* O*_c_* has a crystallite size smaller than that of pure anatase (7.01 vs 8.2 nm), which leads to a compressive effect on the lattice and helps balance the crystallite size, which was expanded by Ce and N elements. The smallest crystallite size is obtained with NP­(Sr) (5.17 nm; Table 1), suggesting that dopants create synergistic effects. Different ionic radii or bonding characteristics of the elements with the anatase phase led to compressive strain, which could restrict the crystallite growth of NP­(Sr).

Reflectance analysis of the single-doped anatase phase was performed to determine the energy band gap (Figure 10b,c). On the basis of the analysis, both nitrogen- and cerium-doped anatase samples could absorb visible light, and both samples exhibited a yellow color. In the case of strontium, the energy gap was 3.3 eV (corresponding to a white color), indicating that doping with strontium increases the energy band gap. The smaller crystallite size of Ti*_a_* Sr*_b_* O*_c_*, compared to that of TiO_2_, is one of the reasons for this increased energy band gap due to the quantum confinement effect.

Cyclic voltammetry of the single-element-doped anatase was carried out to study the effect of the elements on the anatase phase (Figure 10d). The case of Ti*_a_* Ce*_b_* O*_c_* shows a remarkable shift in reduction potential compared to anatase, while in the case of Ti*_a_* N*_b_* O*_c_*, the shift occurred in the oxidation potential. This suggests that cerium modified the conduction band edge of anatase, while nitrogen modified the oxidation band edge of anatase. On the other hand, strontium showed no remarkable change in the oxidation or reduction potential, which was also confirmed by the energy band gap obtained from the reflectance analysis. When Ce^3+^, with an electronic configuration of [Xe]­4f^1^, is doped into TiO_2_, it interacts with the Ti 3d orbitals in the CB and introduces localized electronic states near the conduction band. On the other hand, Ti*_a_* N*_b_* O*_c_* exhibits a modification in the oxidation potential, indicating the hybridization of the 2p orbital of nitrogen with the 2p orbitals of oxygen, which shifts the valence band position.

The surface study was carried out for every single dopant (Figure 10e), and the results were brought in Table 6. As shown, the BET surface area of NP­(Sr) was 105.51 m^2^ g^–1^ (Table 1), whereas it varied for the single-doped samples. The highest surface area was obtained by Ti*_a_* Sr*_b_* O*_c_* (120.25 m^2^ g^–1^), confirming that the strontium dopant can remarkably enhance particle dispersion and surface area. On the other hand, cerium has a medium surface area (100.72 m^2^ g^–1^), which is not very different from the surface area of NP­(Sr) and has no significant effect on the surface area of NP­(Sr). Ti*_a_* N*_b_* O*_c_* exhibited the lowest surface area (38.42 m^2^ g^–1^) compared to NP­(Sr), suggesting that reducing the surface area of the final composite (NP­(Sr)) is beneficial. Regarding the mean pore diameter, Ti*_a_* Sr*_b_* O*_c_* produced the smallest pore diameter (5.27 nm), corresponding to the high surface area (Figure 10f). The same surface area, Ti*_a_* Ce*_b_* O*_c_*, has a medium pore diameter (6.38 nm), which is similar to NP­(Sr), and Ti*_a_* N*_b_* O*_c_* has a pore diameter of 7.03 nm, which confirms its lower surface area and the effect on NP­(Sr). Regarding the pore volume, Ti*_a_* Sr*_b_* O*_c_* showed the highest pore volume (0.44 cm^3^ g^–1^), which helped improve NP’s porosity (Sr). Nitrogen has the smallest pore volume (0.21 cm^3^ g^–1^). These results demonstrated that strontium dopant was the most effective dopant in increasing the surface area of NP­(Sr). In contrast, Ti*_a_* N*_b_* O*_c_* has the most significant effect on reducing the NP­(Sr) surface area. The findings demonstrate how the surface area of NP­(Sr) can be controlled by combining several dopants.

FESEM analysis (Figure S13) showed that all single oxide nanocrystals have irregular shapes except TiO_2_, which is nearly spherical. However, agglomeration can still be observed with TiO_2_ as well (Figure S13a).

### Environment-Based

Some factors, such as pH, temperature, catalyst dosage, reaction time, and dye concentration, are not directly tied to the photocatalyst. Studying how these parameters influence NP­(Sr) efficiency is crucial to progress in photocatalysis and environmental science.

The pH of the environment significantly affected the efficiency of the NP­(Sr) (Table S3). Although reducing different textile dyes was remarkable in neutral conditions, acidic and basic environments could also affect the efficiency. To investigate the role of these properties, different textile dyes, including cationic (methylene blue), anionic (reactive dye, bromophenol blue), and azo anionic (methyl orange), were tested under both basic and acidic environments. It shows that an acidic environment favors the complete removal of anionic textile dyes, while a basic environment favors the cationic dye. The logic behind this phenomenon is due to the positive and negative charges on the neutral surface of NP­(Sr) during the acidic and basic environments, respectively. In an acidic environment, releasing protons (H^+^) makes the NP­(Sr) surface more positively charged, allowing it to absorb anionic textile dyes more effectively. Because the photocatalytic reaction occurs on the surface of the photocatalyst, this reaction is adequately completed by the strong electrostatic force between the charge of the surface of the NP­(Sr) and anionic dyes. This is why methylene blue (cationic dye) is degraded in the basic environment through the negative charge on the NP­(Sr) surface because of the OH^–^ group.

The ζ potential was measured on both bare TiO_2_ and NP­(Sr) to investigate the surface charge behaviors and their relation with dye adsorption (Table 7, Figure S14). The analysis was performed in acidic (pH 4), neutral (pH 7), and basic (pH 11) environments at room temperature. The ζ potential values for pristine TiO_2_ at acidic, neutral, and basic pH levels were measured, and the results follow the expected trend. More positive results were observed as the environment shifted from acidic to neutral and then basic. However, this trend was not observed in NP­(Sr), and surprisingly, the ζ potential is more positive in a basic environment compared to a neutral environment. It is worth noting that the neutral environment means no acidic or basic agents were added. Different reasons might result in this trend, including (I) a shift in the isoelectric point (IEP) through doping. Nitrogen doping as an anionic dopant shifts the IEP toward more acidic values and enhances the negative surface charge density at neutral pH. In addition, Sr^2+^ and Ce^3+^/Ce^4+^ induce lattice strain and the formation of oxygen vacancies, acting as suitable sites for the adsorption of negatively charged species, resulting in (II) double-layer compression and counterion condensation. Under basic conditions, the addition of NaOH leads to an increase in the ionic strength of the suspension. It increases the cation concentration (Na^+^) and results in a reduction of the electrical double layer, which in turn reduces the potential at the slipping plane. (III) synergistic surface passivation. The dopants create a unique surface structure where the high negative charge generated by the nitrogen dopant and vacancy at neutral pH is more stable than in the presence of high salt concentration or competing hydroxyl ions found in basic media.^60–62^

**Table 7:** Results of the ζ Potential Values

sample	ZP (acidic) mV	ZP (neutral) mV	ZP (basic) mV
TiO_2_	–3.15	–3.65	–20.9
NP(Sr)	38.3	–15.2	–6.63

The result also shows that NP­(Sr) has a higher magnitude of surface area than that of pristine TiO_2_. The higher positive potential obtained in an acidic environment for NP­(Sr) confirms stronger surface protonation, which makes it easier to attract anionic dyes, such as bromophenol blue. On the other hand, the more negative potential under neutral conditions enhances surface deprotonation and higher charge density, which improves the adsorption of cationic dyes, such as methylene blue. The result confirms that the dopants enhance the overall surface charge response through lattice strain and defect formation, thereby providing more efficient dye catalyst adsorption under various environmental conditions.^62,63^

Regarding temperature, although the photocatalytic reaction is not very sensitive to temperature, it depends on the type of dye, which can be effective. Generally, temperatures ranging from 20 to 80 °C do not significantly affect the degradation of textile dyes. However, methylene blue showed that increasing the temperature from 30 to 60 °C affects the efficiency in removing textile dyes (Figure 9). The results illustrated that increasing the temperature from 30 to 45 °C leads to better efficiency, although the increase is not significant, while enhancing the temperature from 45 to 60 °C reduces the efficiency. In the range of 30–45 °C, the higher temperature accelerates the reduction of OH^–^, resulting in a higher reduction of methylene blue. However, oxygen levels decline between 45 and 60 °C, resulting in a reduction of one of the primary species responsible for generating free radicals in the photocatalytic dye removal process. This change subsequently impacts overall efficiency.

The efficiency in removing the dye is enhanced by increasing the dosage of the NP­(Sr). We also found this in the range between 0.001 and 0.015 g. However, an observable reduction was between 0.015 and 0.03 g (Figure 8). This reduction is attributed to increased turbidity in the solution, resulting from higher NP­(Sr) dosages and reduced light penetration in the reaction because of greater light scattering.

## Comparative Photocatalytic Performance of the Multidoped TiO_2_ System

The photocatalytic degradation of methylene blue under the best conditions was tested by P25, single dopant, and two-dopant anatases under visible light (Figure S15). The result showed almost no degradation observed by P25, which is because of the properties of TiO_2_ and its energy band gap, which is located in the UV region (Figure S15a). The small decrease in concentration is because MB is a kind of compound that can generate reactive oxygen species (ROS) under the absorption of light. However, degradation is minor compared to using NP­(Sr), and it was around 6% after 2 h.^64,65^ This test confirms that the ability of P25 to degrade MB under visible light is limited. In addition, although MB can generate ROS under light, it is very low under an applied low-intensity LED light. Also, it confirms the photocatalytic box system, which produces visible light in the corresponding region.

On the other hand, multidopant TiO_2_, which were limited to a single or two dopants, were synthesized, including TiSrO, TiNO, TiCeO, TiCeNO, TiSrNO, and TiSrCeO, and compared with NP­(Sr) (Figure S15b–e). It illustrates that although both single- and two-dopant systems degrade MB under visible light, the degradation is much lower compared to that of NP­(Sr), which is able to degrade fully to 100% (Table S11). This behavior is related to the difference in visible-light absorption efficiency and charge-carrier utilization under low-intensity LED illumination.

Although two-dopant photocatalysts can absorb visible light, the photogenerated electrons are not utilized efficiently for the interfacial redox reaction, which is likely due to the less effective charge separation and transport. The results confirm that only narrowing the band gap alone is not sufficient to achieve high photocatalytic activity under low-intensity light, and it is required to design a synergistic system, which can appropriately enhance light absorption, charge separation, and charge transfer to have efficient photocatalytic performance.

## Proposed Mechanism

The combination of Mott–Schottky (MS) and cyclic voltammetry (CV) provides valuable information about the electronic structure of TiO_2_ and NP­(Sr), as a modified sample (Figure 11a,b). The electronic structure and charge transfer behavior of photocatalysts, such as semiconductor type (n- or p-type), donor density (*N*_D_), flat-band potential (*V*_fb_), and the strength of the built-in electric field in the space-charge layer, can be investigated by using MS. On the other hand, redox-active surface states and trap-mediated processes, kinetic shifts caused by varying defect concentrations, electrochemical band-edge positions (effective VB/CB), and confirmation and refinement of band alignment inferred can be studied using optical (DRS) data and cyclic voltammetry.

**Figure 11:**
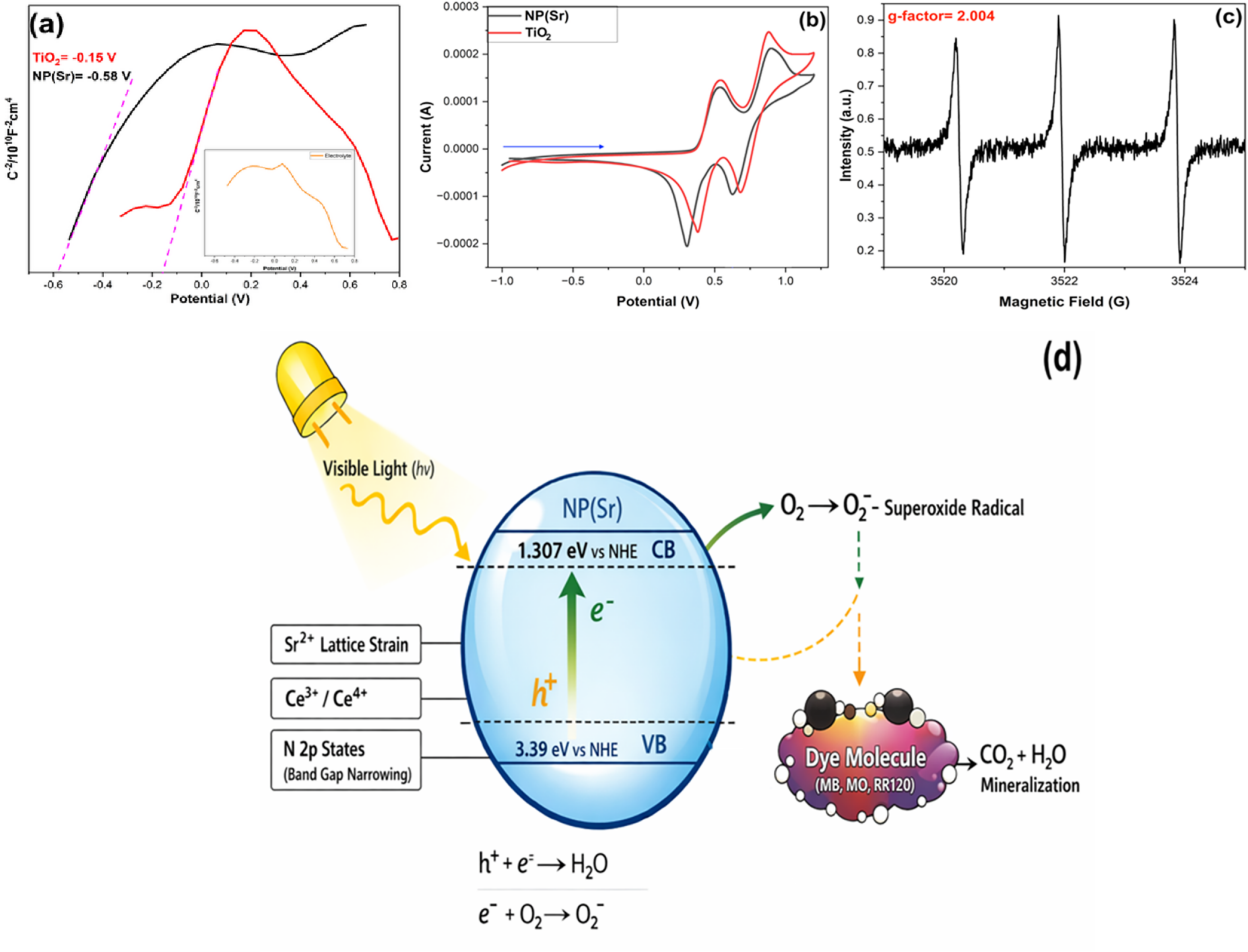
(a) Mott–Schottky, (b) CV analysis, (c) EPR analysis of NP­(Sr), and (d) proposed mechanism for removing textile dyes from water.

The MS plots (*C* ^2–^ vs *potential*) confirm that TiO_2_ is an n-type compound, as indicated by its positive slope. This is not surprising Information, as it has already been proven. In addition, the plot of MS was also located in a more positive area for NP­(Sr), confirming this compound is also an “n-type” photocatalyst (Figure 11a). The result confirms the defect chemistry, where oxygen vacancy (O_v_) and Ti^3+^ centers act as electron donors to TiO_2_. Each element has its own role in making NP­(Sr) an n-type photocatalyst. Sr^2+^ substitutes for Ti^4+^ and produces a charge compensation oxygen vacancy. At the same time, Ce^3+^/Ce^4+^ couples promote O_v_ formation by stabilizing an electron-rich environment. Nitrogen also interacts with the donor defect, modifying the near-edge electronic structure by introducing 2p states. Since both TiO_2_ and NP­(Sr) are n-type compounds, the flat-band potential (*V*_fb_) point is related to the location of the conduction band and located at *V*_fb_ (TiO_2_ = −0.15 V vs Ag/AgCl V, *V*_fb_ (NP­(Sr) = −0.58 V vs Ag/AgCl)). The progressive positive shift in *V*_fb_ from TiO_2_ to NP­(Sr) indicates that the Fermi level moves further from the conduction band upon reconstruction. It is worth noting that the electrolyte was Na_2_SO_4_, and its plot was obtained (inserted figure), which shows a negative slope. Also, the deviation from linearity at higher applied potentials in TiO_2_ is related to the contribution of surface states and nonideal capacitive behavior at the semiconductor–electrolyte interface. Therefore, only the linear region in the depletion regime was used for the determination of the flat-band potential.

The donor density (*N*_D_) is another piece of information that can be obtained from the MS lines. Although the crystallite size of NP­(Sr) is small (even smaller than that of pristine TiO_2_), the very low apparent band gap arises mainly from a high density of deep and localized defect states, such as oxygen vacancies, Ti^3+^, and strong surface disorder, which is related to the irregular morphology of this compound. However, most of these deep traps do not contribute to the mobile carrier density, so they do not translate directly into high *N*_D_.

The conduction band of NP­(Sr) is located at −0.383 V vs normal hydrogen electrode (NHE). With a band gap of 2.71 V (equivalent to 2.71 eV), the valence band position can be calculated through the following eq 4, and the result is observable in Table 8

4 $$E_{\mathrm{VB}}=E_{\mathrm{CB}}+E_{g}$$

**Table 8:** Key Electronic Parameters from DRS, Mott–Schottky, and CV^a^

sample	type	*E*_CB_ (V vs NHE)	*E*_VB_ (V vs NHE)	band gap (eV)
TiO_2_	n-type	+0.047	+3.237	3.19
NP(Sr)	n-type	–0.383	+2.327	2.71

a *E*_NHE_ = *E*_Ag/AgCl_ + 0.197 V.

The surface redox behavior and charge transfer characteristics of the samples were studied by cyclic voltammetry (CV) (Figure 11b). The cathodic response is almost identical for both TiO_2_ and NP­(Sr), indicating that the Ti^4+^/Ti^3+^ reduction process remains unaffected mainly by doping. On the other hand, an observable shift can be seen from TiO_2_ to NP­(Sr) in the anodic region, confirming a modification in hole transfer kinetics and the density of surface states through doping.

The combination of information obtained from the CV and DRS illustrates that the narrowing band gap is not related to crystallite shrinkage but instead to the formation of midgap edge states due to the introduction of dopants. In addition, the oxidation shift confirms that NP­(Sr) contains an abundant deep trap that reduces the energy band gap.

Electron paramagnetic resonance (EPR) was carried out to confirm the qualitative generation and type of radicals (Figure 11c). Three very equal lines made up the EPR spectrum of such a radical caused by the hyperfine interaction (HFI) between an unpaired electron and two equivalent nuclei. Thus, it is possible to indirectly detect the generation of radicals in sample suspensions by comparing the EPR spectra obtained under visible-light irradiation. EPR spectra of materials during laser irradiation are displayed in Figure 11c. The EPR spectra of Tiron at *g* = 2.004 are observed under irradiation, which confirm the presence of the superoxide radicals. It comprises three almost equal lines with a splitting of ∼2 G. The EPR spectra yielded the following HFI constants: HFI1 = 3.6 Gs and HFI2 = 1.9 Gs. EPR spectra exhibit hyperfine splitting consistent with superoxide radical formation, confirming that ^•^O_2_^–^ is the dominant reactive species under visible‑light irradiation.

The intensity of the EPR spectra increases noticeably upon laser irradiation. Tiron radicals are produced when Tiron combines with the produced superoxide (^•^O_2_^–^). However, the primary reason for the increase in the EPR signal under radiation is the production of superoxide in the sample suspensions and its reaction with Tiron.

We detected the EPR spectra from two distinct radicals at pH values higher than 9 in the suspensions. The first radical has a spectrum at *g* = 2.004. There are two lines in the second radical’s spectrum, which are at *g* = 2.005. The interaction of one unpaired electron with one proton and the HFI value of 4.6 Gs results in the formation of such a spectrum.

Therefore, when exposed to a 415 nm laser, the EPR spectra verified that the sample exhibited good efficiency in generating the superoxide anion radical ^•^O_2_^–^. A technique similar to the one described was employed, and it showed that when NP­(Sr) is exposed to visible light, Ti^3+^ is formed due to electrons moving within the Ti–O cluster. Ti^3+^ oxidizes to Ti^4+^, and O_2_^–^ is produced due to interaction with molecular oxygen (O_2_). The exact mechanism underlies the production of ^•^O_2_^–^ in NP­(Sr) when exposed to radiation. The effectiveness of the suggested electron transfer process to an oxygen molecule can be assessed using the EPR method. More superoxide is produced, and an electron is transferred to the oxygen molecule more efficiently when the EPR line intensity is higher.

On the basis of the mentioned studies, the mechanism of the photocatalytic reaction was investigated (Figure 11d). The MS and DRS analyses confirmed the locations of the valence and conduction bands as well as the energy band gap. Then, the EPR showed that the type of radical is the superoxide. Superoxide radicals are formed through the reaction between oxygen and electrons that are excited in the conduction band. The results obtained from testing different temperatures for the photocatalytic reactions also confirmed this. This illustrated that the photocatalytic efficiency was reduced by increasing the temperature and reducing the amount of oxygen.

Additionally, the CV analysis revealed a shift in the conduction peak, which supports this mechanism. This band is actively involved in the electron transfer process. The CB is involved in the reaction because it is located at a range (based on the MS analysis), which is higher than the reduction potential of oxygen (−0.33 V), so it can easily reduce O_2_. However, VB is located at a range, which is higher than 1.23 V (for H_2_O oxidation to O_2_ or, ^•^OH), and it cannot oxidize water. Based on the mentioned discussion, the mechanism of the photocatalytic reaction is proposed in Figure 11d. The details of the mechanism are explained step by step in the following:

5 $$\mathrm{NP}\left(\mathrm{Sr}\right)+h\nu \to e^{-}\left(\mathrm{CB}\right)+h^{+}\left(\mathrm{VB}\right)$$

6 O2+e−(CB)→O•2−

7 O•2−+MB→intermediates

8 radicalintermediates+O•2−→phenothiazinederivatives orsulfonatedaromatics

9 phenothiazinederivatives+O•2−→formicacid,aceticacid,oxalicacid

10 carboxylicacids+O•2−→CO2+H2O

## Predicted Atomic Structure and Defect Formation Mechanism

The result of the experiments confirms that the dopant induces an atomic rearrangement in the anatase lattice, while the overall structure of the anatase remains unchanged. The XRD confirms that all anatase peaks shift to a higher angle after introducing dopants, confirming the lattice contraction. Among all compounds, NP­(Sr) has the smallest crystallite size (5.17 nm), which signals that Sr^2+^ incorporation remarkably reduces the crystallite growth, which is the reason for lattice distortion resulting from ionic radius differences (Sr^2+^ = 1.18 Å vs Ti^4+^ = 0.61 Å).

This mismatch introduces local strain and oxygen vacancies (O_v_) to achieve charge neutrality, which is consistent with the enhanced specific surface area (120 m^2^ g^–1^) and smaller mean pore size (5.27 nm) observed in the BET analysis, both of which are indicative of increased surface defect density.

FT-IR peaks at 1058 and 1368 cm^–1^ are related to the N–O stretching vibration, confirming that intestinal nitrogen is incorporated within the anatase framework and not on the surface, which is also supported by the XPS analysis, where the N 1s peak was not detected on the surface and resides within the bulk lattice. Such interstitial N incorporation shifts the valence band upward via N 2p–O 2p hybridization, as confirmed by cyclic voltammetry, which showed a clear modification of the oxidation potential for N-doped anatase.

On the other hand, Ce dopant modifies the conduction band, and its presence was confirmed through XPS Ce 3d doublet at 881.36 eV, which confirmed the presence of Ce^3+^, whose 4f^1^ electron interacts with Ti 3d orbitals to introduce localized states near the conduction band minimum, enhancing visible-light absorption and electron mobility. The coexistence of Ce^3+^/Ce^4+^ species suggests reversible charge transfer and stabilization of adjacent Ti^3+^ centers and V_o_ sites. The incorporation of Sr introduces a Sr^2+^–O_v_ defect complex, which matches the O 1s symmetry at 532.9 eV and is associated with a high surface area, confirming oxygen deficiency and lattice strain. It is also supported by HRTEM analysis, which confirms the lattice fringes with an interplanar spacing of 0.35 nm corresponding to the anatase plane (101).

The uniform dopant dispersion observed in HAADF-STEM images, along with bright atomic contrast regions, confirms the successful integration of heavier dopants (Sr, Ce) into Ti sites rather than surface segregation.

The electronic and optical data complement these structural observations. The reduction in band gap to 2.71 eV illustrates the defect-mediated modification of the band edge. Cyclic voltammetry results confirmed that Ce^3+^ alters the conduction band edge. At the same time, N doping shifts the valence band, which demonstrates a synergistic modification of the electronic structure consistent with the formation of Ti^3+^–V_o_–Ce^3+^ complexes.

These combined substitutions and interstitial incorporations form defect complexes (Ti^3+^–V_o_–Ce^3+^–Sr^2+^–N) that distort the TiO_6_ octahedra, reduce symmetry, and create localized midgap states. Such structural and electronic rearrangements explain the high charge separation efficiency and visible-light activity of NP­(Sr) (Figure 12).

**Figure 12:**
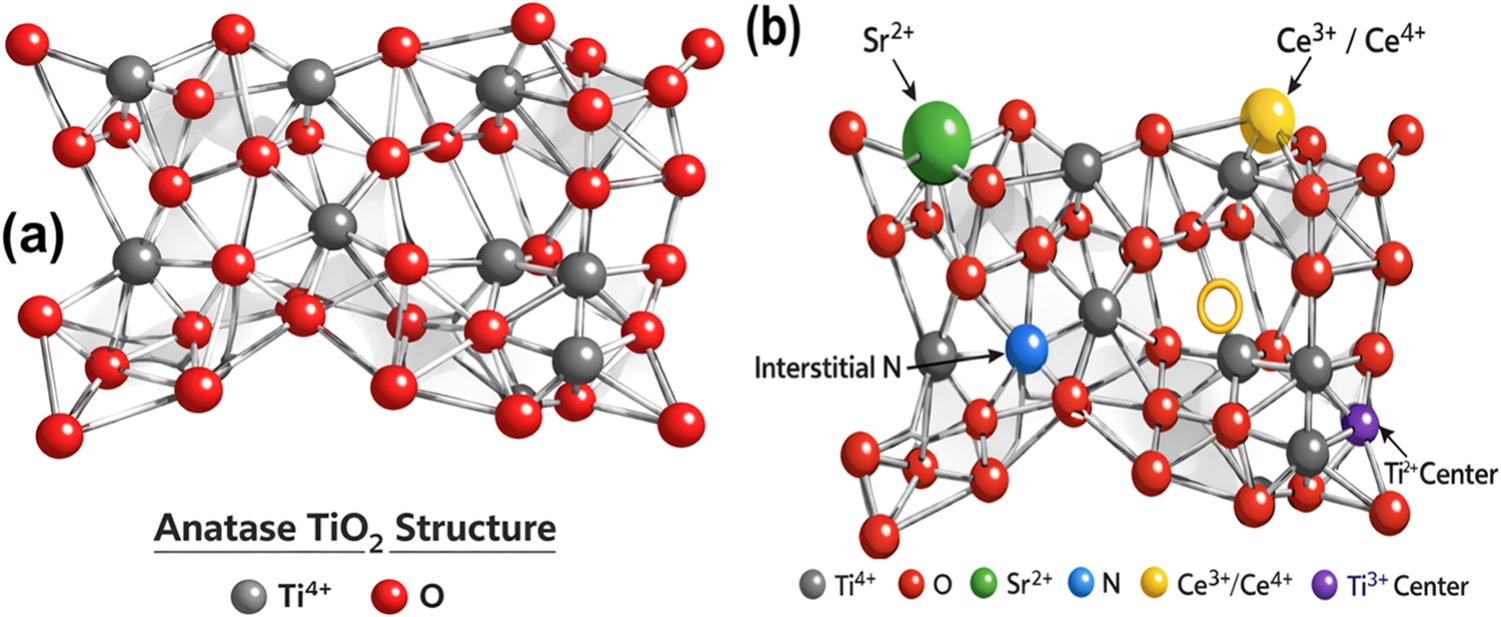
(a) Crystal structure of pristine anatase TiO_2_ illustrating the ordered arrangement of Ti^4+^ (gray) and O^2–^ (red) atoms forming TiO_6_ octahedra. (b) Defect-engineered anatase TiO_2_ lattice showing substitutional doping of Sr^2+^ (green) and Ce^3+^/Ce^4+^ (yellow) at Ti sites, interstitial nitrogen (blue) embedded within TiO_6_ coordination space, Ti^3+^ centers (purple) formed via charge compensation, and an oxygen vacancy (V_o_, yellow ring). The dopant configuration introduces lattice strain, orbital hybridization, and defect complexes, enhancing electronic conductivity and visible-light responsiveness

## Stability of Photocatalyst (NP(Sr))

One of the most important parameters in the catalyst area is their stability for use in multiple cycles. Np­(Sr) stability was tested using five cycles, and each time, the sample was centrifuged and washed with ethanol (Figure S16). The result showed that the crystallite structure of NP­(Sr) in the fifth cycle was maintained as in the first cycle, with a slight shift to the left (Figure S16a). The stability of NP­(Sr) can be related to different reasons, including (a) the strong anatase framework stabilized by Sr^2+^ lattice strain, (b) the reversible Ce^3+^/Ce^4+^ redox coupling that mitigates charge accumulation, and (c) nitrogen incorporation that enhances the electronic uniformity and prevents photocorrosion. In addition, FESEM in accompanied by EDS was carried out after five cycles of degradation to study the morphology of the surface (Figure S16b,c). The result showed no remarkable change on the surface, and still, the shape (sheet-like shape) is the same as NP­(Sr) before photocalytic degradation. It is worth noting that the photocatalyst was used for five different cycles (each 2 h), and each time it was washed, dried, and reused for the next test. In addition, the EDS test illustrates the presence of the corresponding element with almost the same amount, which confirms that no leaching happened.

These results collectively confirm that NP­(Sr) is a durable, reusable, and low-energy photocatalyst suitable for scalable wastewater-treatment applications.

Potential Sr leaching is an important consideration because of environmental and health concerns. The retained anatase structure after five photocatalytic cycles, together with uniform Sr distribution observed by HAADF-STEM, proves incorporation of Sr^2+^ into the TiO_2_ lattice rather than surface segregation. The strong lattice binding is expected to significantly limit the Sr leaching under the studied conditions.

## Comparison of Photocatalytic Efficiencies

The performance of NP­(Sr) was compared with a range of photocatalysts reported in the previous literature (Table S12). The results showed that traditional photocatalysts based on TiO_2_ and ZnO typically require high catalyst dosages (0.5–4 g L^–1^), intense UV radiation, or prolonged reaction times (>3 h) to achieve partial degradation. While NP­(Sr) could completely degrade methylene blue, bromophenol blue, methyl orange, and a reactive dye under mild conditions, applying only 0.0075 g under a low-intensity LED light. In addition, while most reported photocatalysts can reduce the concentration of a single system in removal, NP­(Sr) can effectively work across diverse dye classes. Furthermore, NP­(Sr) does not rely on UV or xenon lamps and also demonstrates fast kinetics, making it more practical for real-world applications.

## Economic Consideration and Justification

In addition to photocatalytic performance, the economic feasibility and scalability of photocatalysts are important factors that need to be considered. The present photocatalyst system uses earth-abundant (low-cost) and environmentally benign elements, including nitrogen and cerium. In this system, the use of noble metals such as Au, Ag, Pt, or Pd, which significantly increase material costs and limit large-scale deployment, was avoided. Titanium dioxide itself is one of the most inexpensive and widely produced photocatalysts, and the selected dopants are industrially available at low cost, ensuring that the overall synthesis remains economically viable. Furthermore, the synthesis process is cheap, and it uses moderate temperatures (≤450 °C), short calcination time (45 min), and low-pressure hydrothermal treatment, which collectively reduce energy consumption and production costs compared to common high-temperature or multistep fabrication routes. Most importantly, the ability of NP­(Sr) to operate efficiently under ultralow-intensity LED illumination (9.55 mW cm^–2^) significantly reduces operational energy costs, representing a substantial advantage over traditional photocatalysts that need high-power UV or xenon lamps (Table S13).

### Economic Analysis

We have added a cost comparison table in the Supporting Information. Preliminary estimates show: (1) Raw material cost for NP­(Sr) is approximately USD 45–60/kg (vs USD 25–35/kg for commercial P25 TiO_2_); (2) However, the 10–100 time lower catalyst dosage requirement (0.125 vs 0.5–4 g L^–1^ for conventional photocatalysts) and elimination of UV lamps significantly reduce operational costs; (3) Life-cycle cost analysis indicates NP­(Sr) is economically competitive when considering complete dye mineralization under standard LED lighting.

The objective behind employing a multimetal doping strategy is to achieve synergistic modulation of structural, optical, and electronic properties that cannot be realized through single-element doping. As mentioned before, in this system, cerium (Ce) introduces localized 4f electronic states near the conduction band, enhancing visible-light absorption and modifying electron trapping–detrapping processes. Nitrogen (N) modifies the valence band by N­(2p)–O­(2p) hybridization, which leads to effective band gap narrowing and extended visible-light response. Strontium (Sr), due to its large ionic radius, affects the lattice strain and oxygen vacancy formation, decreasing crystallite growth, enhancing surface area, and improving charge transport and separation. The combination of the effects of dopants creates a cooperative electronic structure and defect landscape, enabling efficient charge carrier generation, separation, and surface reaction kinetics under extremely low photon flux. This rational multidopant design strategy directly helps complete dye degradation under realistic, low-energy LED illumination, representing a substantial advancement toward sustainable, economically viable photocatalytic wastewater treatment technologies.

## Conclusion

This work successfully developed and assessed new four-nuclear oxide nanocrystals, NP­(Sr), as efficient photocatalysts for degrading textile dyes under visible-light irradiation. In this regard, the strategic doping of alkali, alkaline-earth, nitrogen, titanium, and cerium elements in a comprehensive synthesis approach has resulted in marked enhancements of various essential parameters of photocatalytic performance, including energy band gap modulation, increased surface area, decreased electron recombination, improved durability, and enhanced structural stability. This catalyst demonstrated high effectiveness for wastewater purification on an industrial scale, achieving nearly 100% removal efficiency in under 2 h for various dye types, including cationic, anionic, and azo dyes.

Mechanistic studies provided better insight into the superior performance exhibited by NP­(Sr), attributed to efficient electron–hole separation resulting from superoxide radical generation.

It was shown how quantum confinement terminology could affect the crystallite size of NP­(Sr) because of strontium, which consequently affects the large surface area. Therefore, the catalyst showed excellent stability over multiple cycles, indicating its practical applicability.

## Supplementary Material



## Data Availability

The data supporting this article have been included as part of the Supporting Information.

## Notes

Additional experimental design data; structural, morphological, and surface characterization; full photocatalytic degradation and kinetics data for all four dye classes; statistical analyses; comparative photocatalytic performance; and light-source specifications (PDF)

## Abbreviations

NP­(Cs): Ti*_a_* Cs*_b_* Ce*_c_* N*_d_* O*_e_*
NP­(Ca): Ti*_a_* Ca*_b_* Ce*_c_* N*_d_* O*_e_*
NP­(Sr): Ti*_a_* Sr*_b_* Ce*_c_* N*_d_* O*_e_*
NP­(Ba): Ti*_a_* Ba*_b_* Ce*_c_* N*_d_* O*_e_*
NP­(Na): Ti*_a_* Na*_b_* Ce*_c_* N*_d_* O*_e_*
NP­(Mg): Ti*_a_* Mg*_b_* Ce*_c_* N*_d_* O*_e_*
NP­(Rb): Ti*_a_* Rb*_b_* Ce*_c_* N*_d_* O*_e_*
Ti*_a_* M*_b_* Ce*_c_* N*_d_* O*_e_*: M is different metals, including Cs, Ca, Sr, Ba, Na, Mg, Rb
