# Supplementary material for: Efficient Photocatalytic Degradation of Textile Dyes Using Four-Element Doped Anatase Nanocrystals under Low-Intensity LED Light
Source: ACS Omega. 2026 Jun 2;11(23):33743–62. doi: 10.1021/acsomega.6c00166 (PMC13281005; doi:10.1021/acsomega.6c00166)
Supplement: Supplementary file 1 [file ao6c00166_si_001.pdf]

Efficient Photocatalytic Degradation of Textile Dyes Using Four Element DopedAnatase nanocrystals Under Low-Intensity LED Light

Yahya Absalan*[1], Abhyuday Mandal[2], Ramaraja Pandian Ramasamy[3], Baviththira Suganthan[3], Yehia Khalifa[4], and Suraj Sharma*[5]

[1]Department of Chemistry, University of Georgia, Athens, Georgia 30602, United States
[2]Department of Statistics, University of Georgia, Athens, Georgia 30602, United States
[3]Nano Electrochemistry Laboratory, School of Chemical, Materials and Biomedical Engineering, College of Engineering, University of Georgia, Athens, GA 30602, USA
[4]College of Arts and Sciences Department of Chemistry and Biochemistry, The Ohio State University, Columbus, Ohio 43210, USA
[5]Department of Textiles, Merchandising, and Interiors, University of Georgia, Athens, Georgia 30602, United States

Content

Table S1. Experimental design matrix (81 conditions) used to optimize photocatalytic degradation parameters, including pH, dye concentration, catalyst dosage, and temperature, with corresponding dye removal efficiencies.
Figure S1. Original XRD of the photocatalysts, (b)FTIR spectra of NP(Sr) confirm the presence of surface hydroxyl groups and nitrogen incorporation, indicative of successful multi-doping and surface modification.
Table S2. Statistical analysis of FESEM measurements for NP(Sr) showing standard deviation and particle size distribution.
Figure S2. FESEM micrographs of the synthesized photocatalysts illustrating sheet-like morphologies influenced by ethylenediamine-mediated synthesis.
Figure S3. Surface area and porosity analysis of NP(Sr), with the inset showing the pore diameter distribution, confirming mesoporosity and increased surface area.
Table S3. Photocatalytic degradation of methylene blue by NP(Sr) under various experimental conditions, listing first-order and real rate constants. Total organic carbon measurement for each sample
Figure S4. Photocatalytic reduction of different photocatalysts under visible light (pH = basic, dye concentration = 0.0167 g L^{-1}, catalyst dosage = 0.0075 g, temperature = 30 °C). (a) Photocatalytic degradation of methylene blue by NP(Sr), (b) photodecomposition efficiency of NP(Sr), and (c) photocatalytic degradation kinetics of NP(Sr) under tube lamps
Figure S5. Kinetic profiles for methylene blue degradation under visible light showing (a) different pH, (b) dye concentrations, (c) catalyst dosages, and (d) temperatures
Figure S1. Comparison of the reduction in concentration of methylene blue under different pH conditions: (a) acidic, (b) neutral, (c) basic. (d) photodecomposition efficiency of NP(Sr) under visible light
Figure S2. Comparison of the reduction in concentration of methylene blue under different dye concentrations: (a) 0.0083 gL^{-1}, (b) 0.0167 gL^{-1}, (c) 0.0333 gL^{-1}. (d) photodecomposition efficiency of NP(Sr) under visible light

Figure S3. Comparison of the reduction in concentration of methylene blue under different dosages of NP(Sr): (a) 0.001g, (b) 0.0075g, (c) 0.015g, (d) 0.03g, (e) photodecomposition efficiency of NP(Sr) under visible light
Figure S4. Comparison of the reduction in concentration of methylene blue under different temperatures: (a) 30℃, (b) 45℃, (c) 60℃, (d) photodecomposition efficiency of NP(Sr) under visible light, (e) The second run of the photocatalytic degradation under visible wavelength by NP(Sr), and (f) The third run of the photocatalytic degradation under visible wavelength by NP(Sr)
Table S4. Degradation of bromophenol blue (BPB) by NP(Sr) under visible light, with first-order rate constants and R^2 correlation values
Figure S10. Photocatalytic degradation of bromophenol blue under (a) acidic, (b) neutral, and (c) basic conditions, with (d) efficiency comparison and (e) kinetic plots
Table S5. Photocatalytic degradation of methyl orange (MO) by NP(Sr) under visible light, showing kinetic rate constants and correlation coefficients
Figure S11. Photocatalytic degradation of methyl orange in (a) acidic, (b) neutral, (c) basic media, with (d) photodecomposition efficiency and (e) kinetic fits.
Table S6. Degradation kinetics of reactive red 120 (RR120) by NP(Sr) under visible light, listing rate constants and R^2 values.
Figure S12. Photocatalytic degradation of reactive red 120 in (a) acidic, (b) neutral, (c) basic environments, showing (d) decomposition efficiency and (e) kinetic modeling results.
Figure S13. Morphology study of single dopants by FESEM; (a) TiO2, (b) $Ti_{1-y}Ce_yO_{2-\delta}$, (c) $TiO_{2-\delta}N_z$, and (d) $Ti_{1-x}Sr_xO_{2-\delta}$
Figure S14. Zeta potential of TiO_2 in (a) acidic (PH 2), (b) neutral, and (c) basic (PH 11), and NP(Sr) in (d) acidic (PH 2), (e) neutral, and (f) basic (PH 11)
Table S7. Coefficients for the natural environment (pH=7)
Table S8. Coefficients for the natural environment (pH<7)
Table S9. Coefficients for the natural environment (pH>7)
Table S10. Test of different temperatures (3 repetitions for each)
Table S11. Compare the degradation of methylene blue by different multi-dopant photocatalysts under visible light
Figure S15. (a) Photocatalytic degradation of methylene blue by (a) TiO_2 (P25), (b) TiSrNO, (c) TiCeNO, (d) TiSrCeO, (e) TiSrO (f) TiNO, (g) TiCeO, (h) photodecomposition efficiency of different multi-doped anatase, and (i) photocatalytic degradation kinetics of the nano-photocatalysts under visible light
Figure S16. *Stability test of the photocatalyst over five consecutive photocatalytic cycles.*
Table S12. Comparative study of the photocatalytic efficiencies of the different photocatalysts
Table S13. Characteristics of light sources used in photocatalytic studies, including LEDs, xenon lamps, and natural sunlight, showing power, spectral distribution, and intensity.

Table S1. Experimental design matrix (81 conditions) used to optimize photocatalytic degradation parameters, including pH, dye concentration, catalyst dosage, and temperature, with corresponding dye removal efficiencies.

Row	PH	Concentration of Dye (g)	Dosage of Catalyst (g)	Temperature (°C)	Concentration of dyes after reaction (mol)	Percentage of dye left
1	Basic	0.000334	0.01	60	0.03056	3.02%
2	Basic	0.000166	0.0025	60	0.04282	4.23%
3	Basic	0.000166	0.005	30	0.05	4.94%
4	Basic	0.000166	0.01	60	0.05037	4.98%
5	Basic	0.000166	0.005	60	0.06438	6.36%
6	Basic	0.000084	0.0025	60	0.06635	6.56%
7	Basic	0.000084	0.01	45	0.072	7.11%
8	Basic	0.000334	0.0025	60	0.12289	12.14%
9	Basic	0.000166	0.01	45	0.18641	18.42%
10	Basic	0.000084	0.0025	45	0.22361	22.10%

11	Basic	0.000166	0.0025	45	0.28209	27.88%
12	Basic	0.000334	0.005	60	0.31329	30.96%
13	Acidic	0.000334	0.005	30	0.35623	35.20%
14	Basic	0.000166	0.005	45	0.38551	38.10%
15	Neutral	0.000166	0.01	30	0.459	45.36%
16	Acidic	0.000166	0.005	30	0.47503	46.94%
17	Basic	0.000166	0.0025	30	0.48365	47.81%
18	Acidic	0.000334	0.01	30	0.52022	51.39%
19	Basic	0.000166	0.01	30	0.57774	57.09%
20	Basic	0.000334	0.005	45	0.60198	59.50%
21	Neutral	0.000084	0.005	45	0.67101	66.29%
22	Neutral	0.000166	0.0025	45	0.693	68.46%
23	Neutral	0.000166	0.005	30	0.71	70.15%
24	Neutral	0.000166	0.01	45	0.748	73.91%
25	Neutral	0.000084	0.01	45	0.74951	74.06%
26	Basic	0.000334	0.0025	30	0.75473	74.56%
27	Neutral	0.000166	0.005	45	0.756	74.68%
28	Neutral	0.000084	0.0025	60	0.77138	76.23%
29	Basic	0.000334	0.01	45	0.77363	76.46%
30	Basic	0.000084	0.005	45	0.81	80.05%
31	Basic	0.000084	0.0025	30	0.81	80.05%
32	Neutral	0.000084	0.005	30	0.81223	80.27%
33	Acidic	0.000084	0.01	45	0.81757	80.82%
34	Acidic	0.000084	0.01	60	0.81757	80.82%
35	Basic	0.000334	0.01	30	0.81992	81.06%
36	Neutral	0.000084	0.01	60	0.827	81.75%
37	Neutral	0.000084	0.0025	30	0.8288	81.93%
38	Neutral	0.000334	0.0025	30	0.8299	82.04%
39	Basic	0.000084	0.005	60	0.84	83.01%
40	Neutral	0.000334	0.005	30	0.84447	83.45%
41	Basic	0.000084	0.01	30	0.86	84.98%
42	Acidic	0.000166	0.01	45	0.86088	85.07%
43	Neutral	0.000166	0.0025	60	0.866	85.61%
44	Neutral	0.000084	0.01	30	0.88077	87.03%
45	Neutral	0.000166	0.0025	30	0.882	87.15%
46	Neutral	0.000166	0.005	60	0.888	87.74%
47	Acidic	0.000166	0.01	30	0.90154	89.08%
48	Neutral	0.000334	0.01	45	0.9177	90.68%
49	Acidic	0.000084	0.005	30	0.9203	90.94%

50	Acidic	0.000334	0.0025	30	0.92045	90.95%
51	Acidic	0.000166	0.0025	60	0.92292	91.19%
52	Neutral	0.000334	0.01	60	0.92379	91.28%
53	Neutral	0.000334	0.0025	45	0.9256	91.46%
54	Neutral	0.000334	0.005	45	0.92825	91.74%
55	Acidic	0.000166	0.005	60	0.92947	91.86%
56	Acidic	0.000084	0.005	45	0.93079	91.98%
57	Neutral	0.000334	0.005	60	0.93519	92.40%
58	Acidic	0.000166	0.005	45	0.93932	92.80%
59	Acidic	0.000166	0.01	60	0.94141	93.00%
60	Acidic	0.000334	0.01	60	0.94323	93.17%
61	Acidic	0.000084	0.0025	60	0.94941	93.78%
62	Neutral	0.000334	0.0025	60	0.94941	93.78%
63	Acidic	0.000084	0.0025	30	0.95795	94.68%
64	Acidic	0.000334	0.01	45	0.96057	94.94%
65	Neutral	0.000166	0.01	60	0.964	95.26%
66	Acidic	0.000084	0.01	30	0.96944	95.75%
67	Neutral	0.000334	0.01	30	0.97214	96.02%
68	Acidic	0.000334	0.005	60	0.97352	96.15%
69	Acidic	0.000334	0.0025	60	0.97371	96.17%
70	Neutral	0.000084	0.005	60	0.9839	97.23%
71	Neutral	0.000084	0.0025	45	0.99378	98.22%
72	Acidic	0.000166	0.0025	45	0.9997	98.80%
73	Acidic	0.000166	0.0025	30	0.99971	98.80%
74	Acidic	0.000334	0.0025	45	1.01186	100.00%
75	Acidic	0.000334	0.005	45	Inconclusive	
76	Acidic	0.000084	0.005	60	Inconclusive	
77	Acidic	0.000084	0.0025	45	Inconclusive	
78	Basic	0.000334	0.005	30	Inconclusive	
79	Basic	0.000334	0.0025	45	Inconclusive	
80	Basic	0.000084	0.005	30	Inconclusive	
81	Basic	0.000084	0.01	60	Inconclusive	

Results marked 'not clear' indicate conditions where UV-Vis analysis did not yield reliable absorbance data, likely due to side product formation or concentration effects

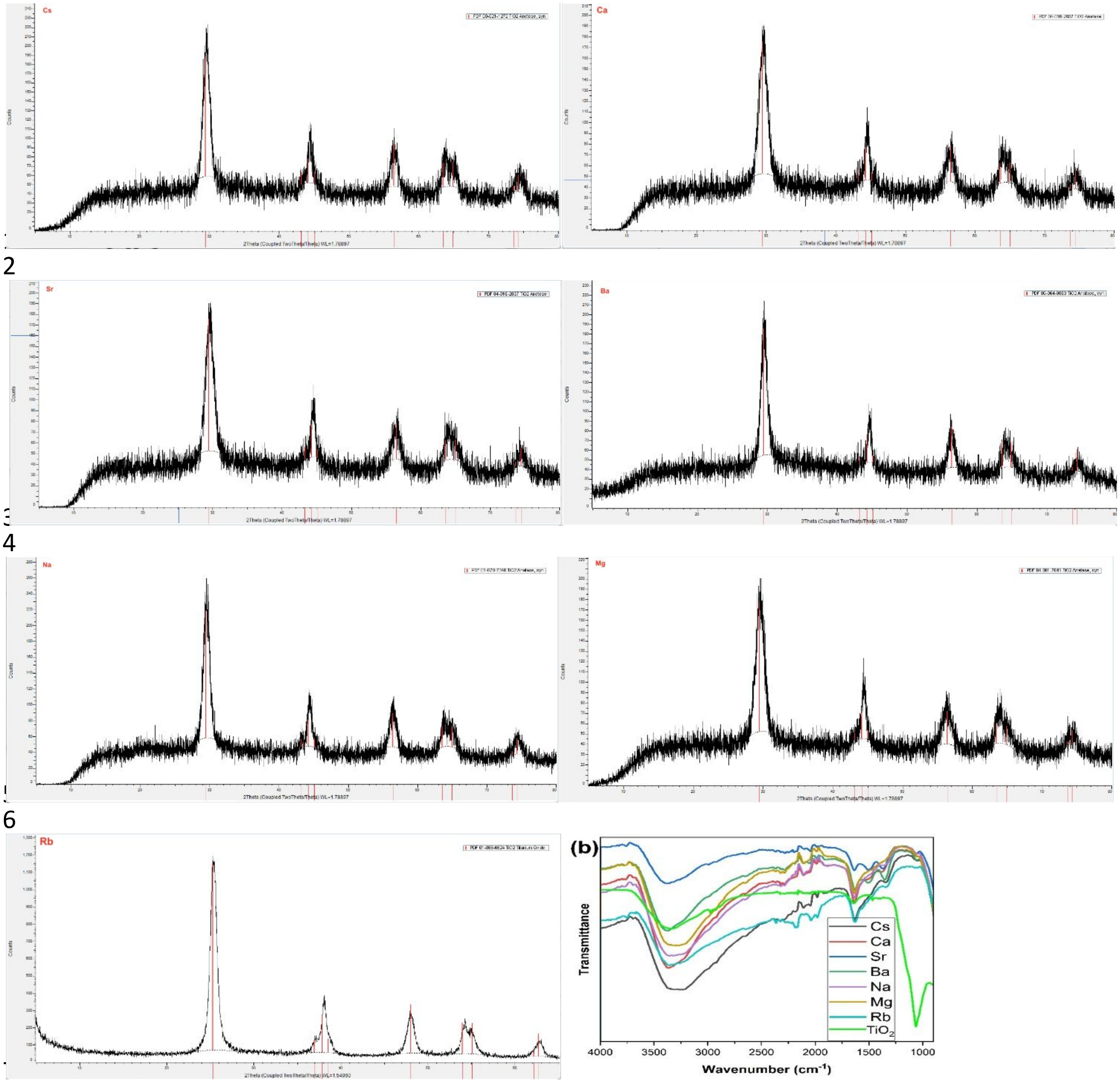

Figure S1. Original XRD of the photocatalysts, (b)FTIR spectra of NP(Sr) confirm the presence of surface hydroxyl groups and nitrogen incorporation, indicative of successful multi-doping and surface modification.

XRD Analysis and Lattice Parameter Calculations

1. d-Spacing Calculation Using Bragg's Law

Bragg's Law: $d = \lambda / (2 \sin \theta)$, where $\lambda = 1.541$ Å (Cu Kα radiation).

Pristine Anatase (101)

$2\theta = 25.3°$

$\theta = 12.65°$

$d_{101} = 3.518$ Å $= 0.352$ nm

NP(Sr) (Shifted Peak)

$2\theta = 29.3°$

$\theta = 14.65°$

$d_{101} = 3.046$ Å $= 0.305$ nm

Percent Contraction

Contraction = $((3.046 - 3.518) / 3.518) \times 100 = -13.4\%$

The Sr-doped sample shows a 13.4% contraction in the (101) interplanar spacing compared to pristine anatase.

2. Lattice Parameter Estimation

Anatase TiO_2 has a tetragonal structure defined by: $1/d^2 = (h^2+k^2)/a^2 + l^2/c^2$.

For the (101) plane: $1/d_{101}^2 = 1/a^2 + 1/c^2$.

Estimated Lattice Parameters (Assuming Isotropic Contraction)

Scaling factor s = 3.046 / 3.518 = 0.866

Literature anatase parameters:

$a_0 = 3.784$ Å

$c_0 = 9.514$ Å

Estimated NP(Sr) parameters:

$a \approx 3.28$ Å

$c \approx 8.24$ Å

These values represent an estimated 13.4% lattice contraction assuming isotropic shrinkage. This magnitude of contraction is unusually large for simple substitutional doping and should be interpreted cautiously.

Table S2. Statistical analysis of FESEM measurements for NP(Sr) showing standard deviation and particle size distribution.

**Number**	**Length of particle (X_i)**	**Mean size**<br>**Mean (μ) = Sum / N**<br>N: number of the data=15	**Squared Differences (X_i - μ)²**	**Variance**<br>**σ² = Σ((Xi - μ)²) / (N - 1)**	**Standard Deviation (σ) = √Variance**

1	26.52	22.53 nm	15.94	19.50 nm²	4.42 nm
2	19.19		11.13		
3	25.57		9.26		
4	25.57		9.26		
5	23.94		1.99		
6	28.36		34.02		
7	21.85		0.45		
8	24.92		5.72		
9	30.53		64.05		
10	21.82		0.49		
11	16.21		39.90		
12	20.23		5.27		
13	15.66		47.15		
14	18.82		13.73		
15	18.71		14.56		

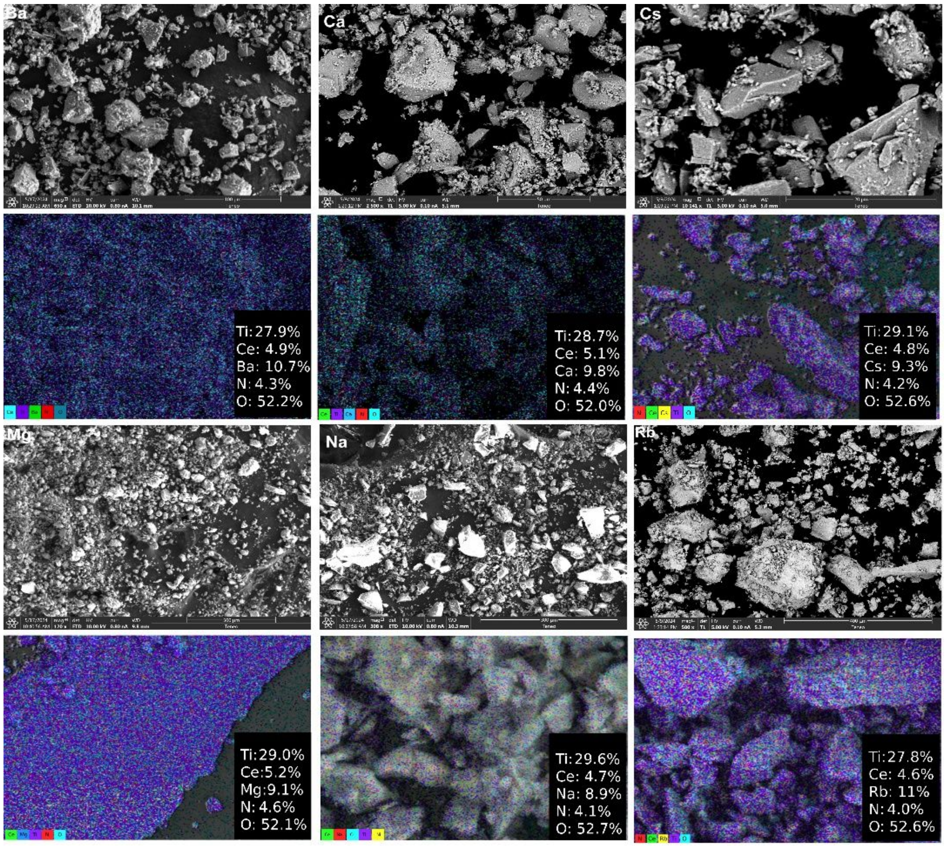

Figure S2. FESEM micrographs of the synthesized photocatalysts illustrating sheet-like morphologies influenced by ethylenediamine-mediated synthesis.

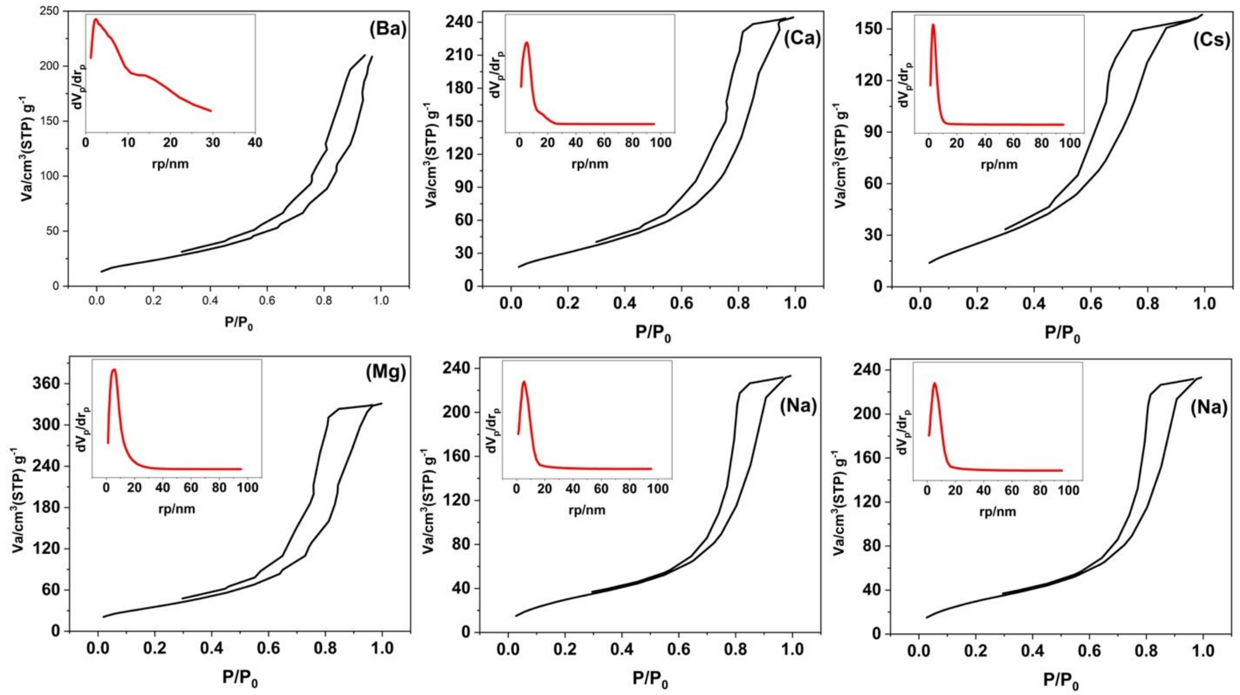

Figure S3. Surface area and porosity analysis of NP(Sr), with the inset showing the pore diameter distribution, confirming mesoporosity and increased surface area.

Table S3. Photocatalytic degradation of methylene blue by NP(Sr) under various experimental conditions, listing first-order and real rate constants. Total organic carbon measurement for each sample

		First-order rate		Real rate	
**Method**	**Degradation (%)**	**Kapp(min^{-1})**	**R^2**	**Kapp(min^{-1})**	**R^2**
**Initial Photocatalytic Comparison**	42%	0.0048x	0.7609	8E-05x^2	1
		**Effect of pH**			
pH (Acidic)	2%	0.0002x	0.9926	*	*
pH (Neutral)	42%	0.0048x	0.7609	8E-05x^2	1
pH(Basic)	82%	0.0114x	0.9472	*	*
		**Effect of Dye Concentration**			
pH: basic, 0.0167 gL^{-1}	82%	0.0028x	0.9005	*	*
pH: basic, 0.0083 gL^{-1}	85%	0.0114x	0.9472	*	*
pH: basic, 0.0333 gL^{-1}	30%	0.0204x	0.8161	0.0003x^2	0.9934
		**Effect of the Catalyst's Dosage**			
pH: basic, dye concentration: 0.0167 gL^{-1}, 0.001g	82%	0.0028x	0.7917	4E-05x^2	0.989
pH: basic, dye concentration: 0.0167 gL^{-1}, 0.0075g	97%	0.0205x	0.8185	0.8185	0.9943
pH: basic, dye concentration: 0.0167 gL^{-1}, 0.015g	97%	0.0202x	0.8092	0.0003x^2	0.9934

pH: basic, dye concentration: 0.0167 gL-1, 0.03g	83%	0.0028 x	0.9005	*	*
**Effect of Temperature**					
pH: basic, dye concentration: 0.0167 gL-1, 0.0075g, 30℃	100%	0.0205 x	0.8185	0.8185	0.9943
pH: basic, dye concentration: 0.0167 gL-1, 0.0075g, 45℃	100%	0.0314 x	0.8013	$0.0005x^2$	0.9913
pH: basic, dye concentration: 0.0167 gL-1, 0.0075g, 60℃	75%	0.0161 x	0.9467	*	*

Total Organic Carbon (TOC)

Sample	Total Organic C (mg/L)	Percentage%	Mineralization achieved (%)
100% MB solution	2.573	100	0.0
15 min exposing by light	2.163	84.1	15.9
30 min exposing by light	1.857	72.2	27.8
45 min exposing by light	1.503	58.4	41.6
60 min exposing by light	1.318	51.2	48.8
75 min exposing by light	0.386	15.0	85.0
90 min exposing by light	0.318	12.4	87.6
105 min exposing by light	0.214	8.3	91.7
100% pure water (Pure water blank)	0.183	7.1	92.9 (not degradation; it's the blank)

*: No need because the accuracy is more than 90%

Initial Photocatalytic Comparison: It is the comparison between photocatalysts before testing different effective parameters

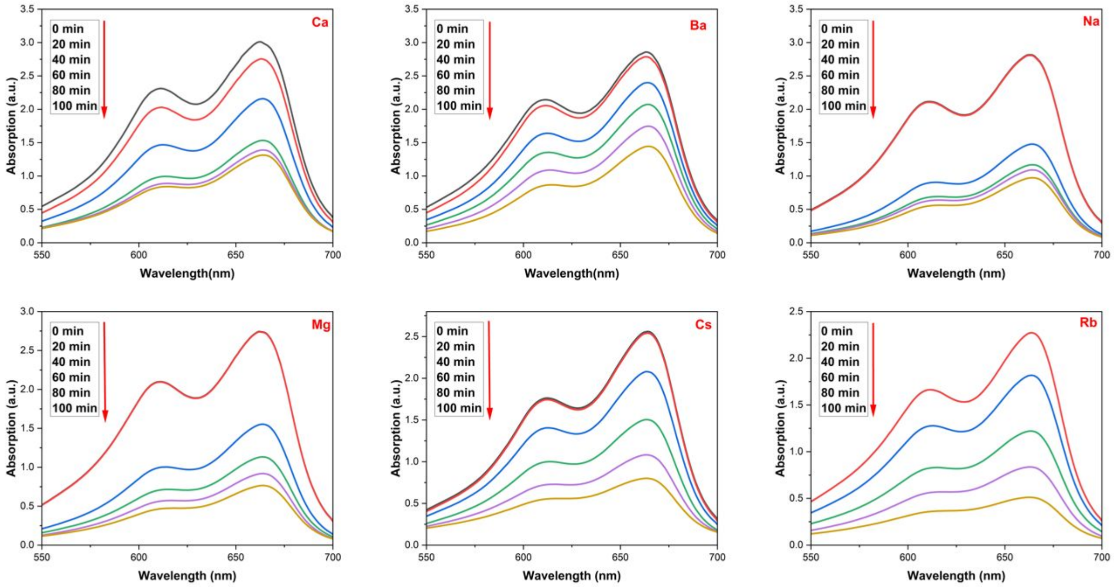

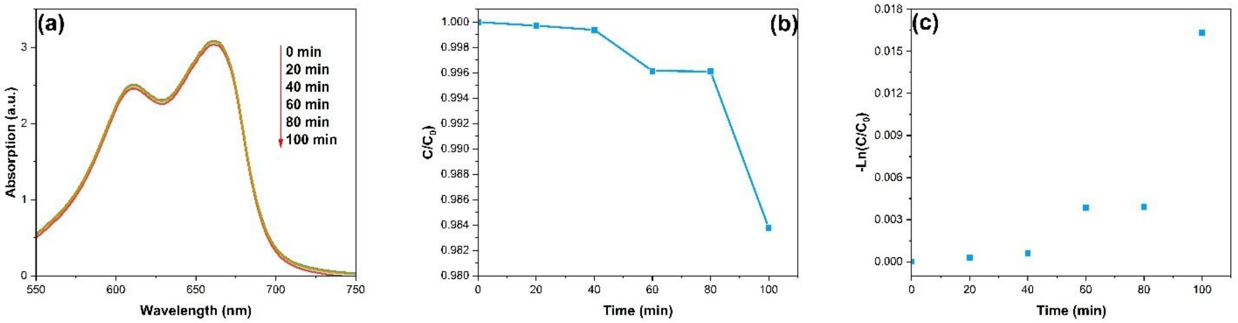

Figure S4. Photocatalytic reduction of different photocatalysts under visible light (pH = basic, dye concentration = 0.0167 g L^{-1}, catalyst dosage = 0.0075 g, temperature = 30 °C). (a) Photocatalytic degradation of methylene blue by NP(Sr), (b) photodecomposition efficiency of NP(Sr), and (c) photocatalytic degradation kinetics of NP(Sr) under tube lamps

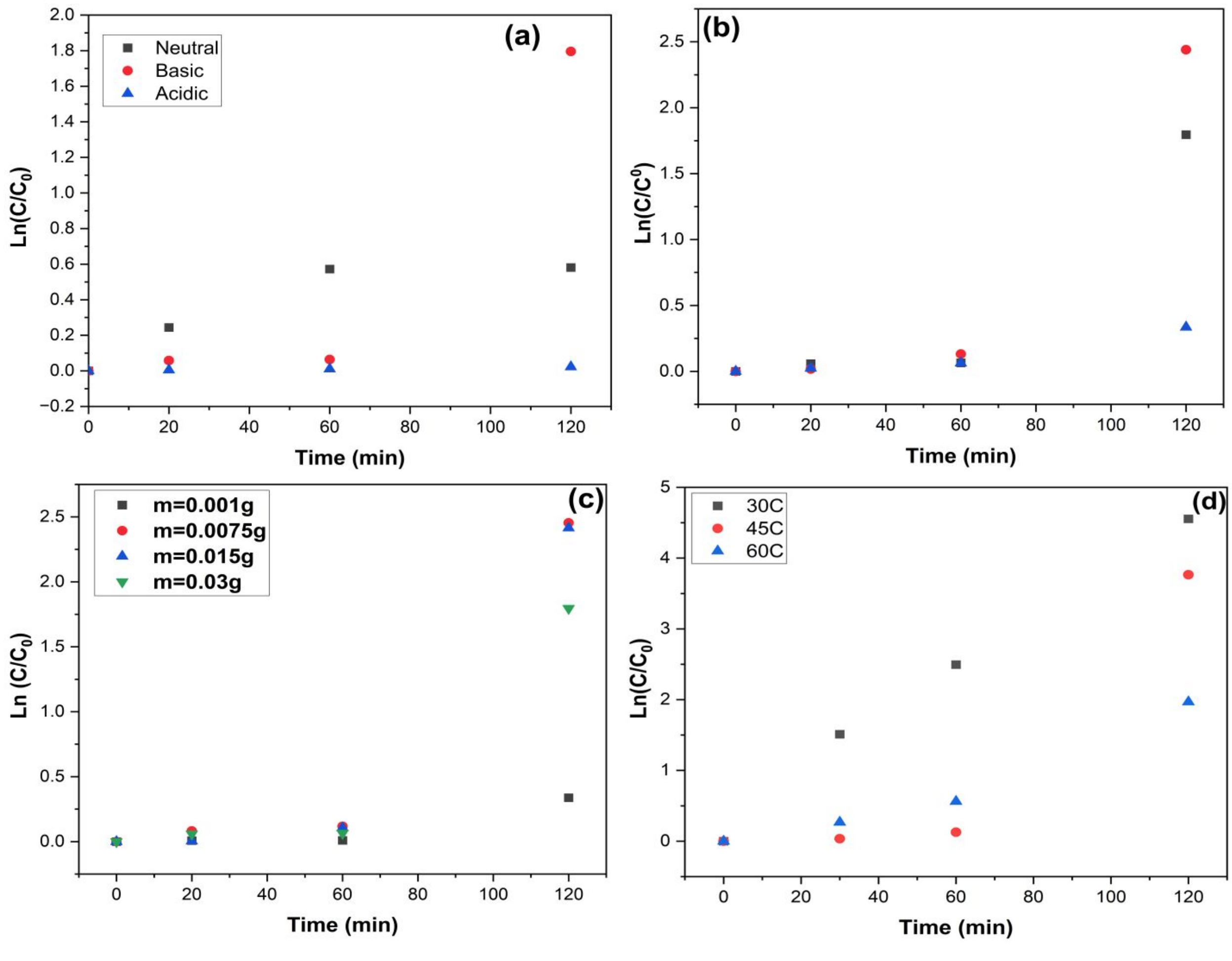

Figure S5. Kinetic profiles for methylene blue degradation under visible light showing (a) different pH, (b) dye concentrations, (c) catalyst dosages, and (d) temperatures

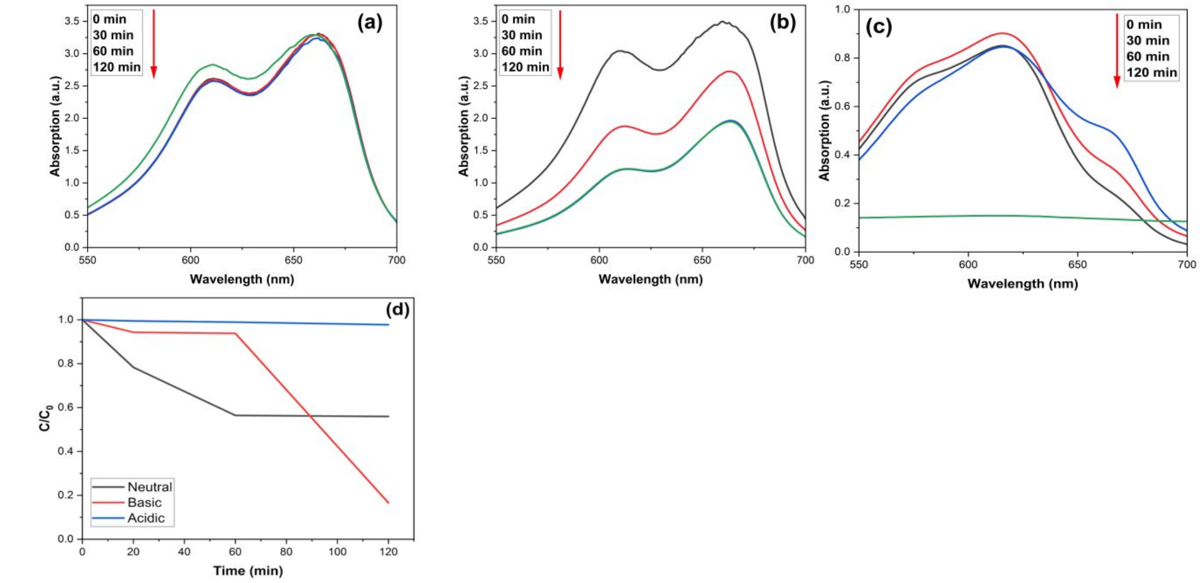

Figure S5. Comparison of the reduction in concentration of methylene blue under different pH conditions: (a) acidic, (b) neutral, (c) basic. (d) photodecomposition efficiency of NP(Sr) under visible light

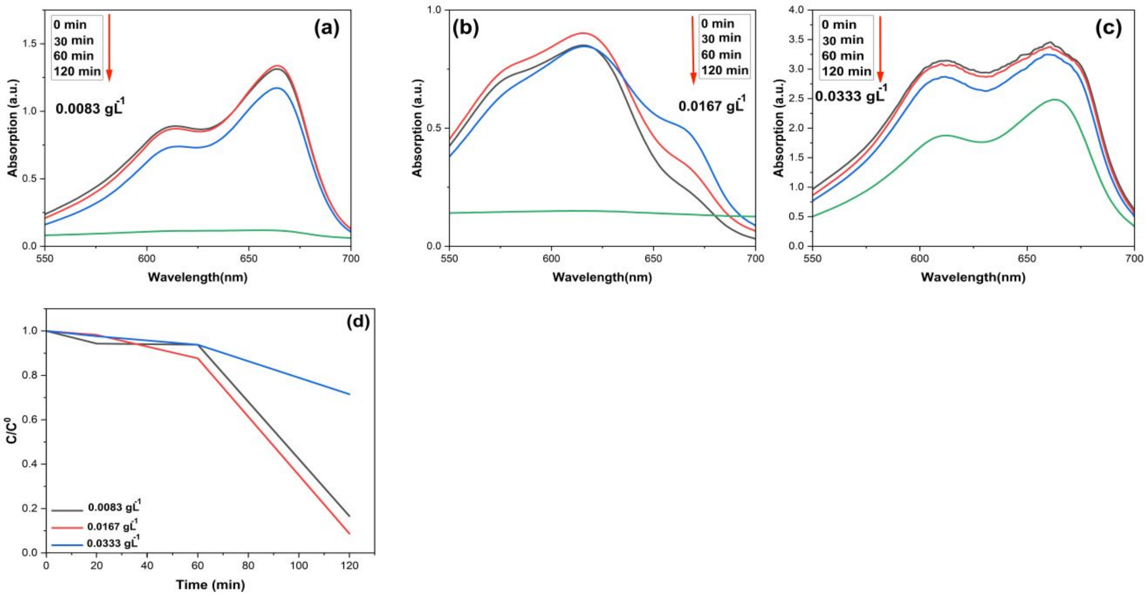

Figure S6. Comparison of the reduction in concentration of methylene blue under different dye concentrations: (a) 0.0083 gL^{-1}, (b) 0.0167 gL^{-1}, (c) 0.0333 gL^{-1}. (d) photodecomposition efficiency of NP(Sr) under visible light

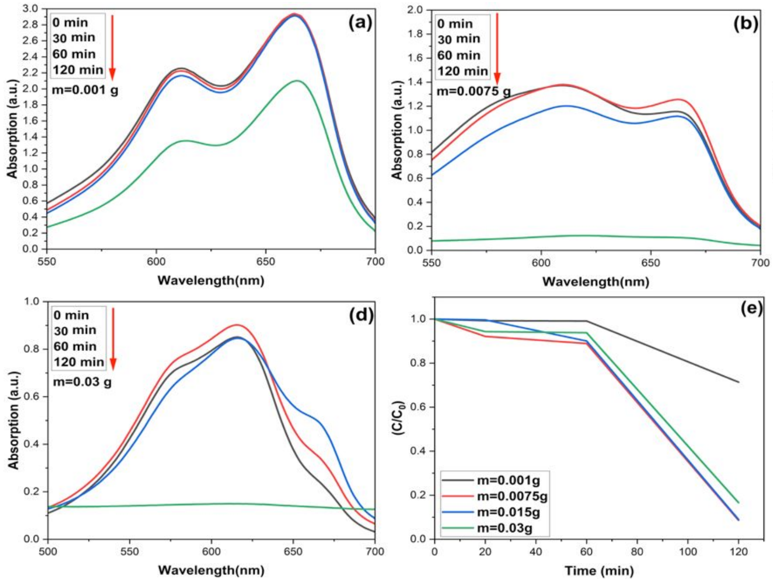

Figure S7. Comparison of the reduction in concentration of methylene blue under different dosages of NP(Sr): (a) 0.001g, (b) 0.0075g, (c) 0.015g, (d) 0.03g, (e) photodecomposition efficiency of NP(Sr) under visible light

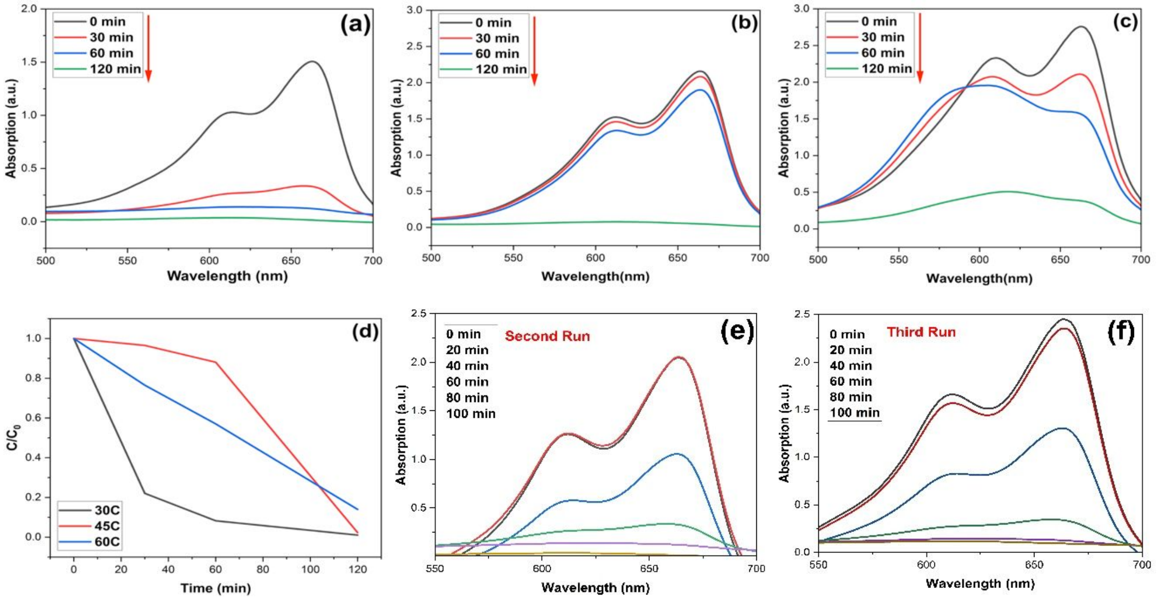

Figure S8. Comparison of the reduction in concentration of methylene blue under different temperatures: (a) 30℃, (b) 45℃, (c) 60℃, (d) photodecomposition efficiency of NP(Sr) under visible light, (e) The second run of the photocatalytic degradation under visible wavelength by NP(Sr), and (f) The third run of the photocatalytic degradation under visible wavelength by NP(Sr)

Table S4. Degradation of bromophenol blue (BPB) by NP(Sr) under visible light, with first-order rate constants and R² correlation values

	First-order rate		Real rate	
Degradation (%)	$K_{app}(min^{-1})$	R^2	$K_{app}(min^{-1})$	R^2

**pH(acidic)**	90	0.0239x	0.9029	*	*
**pH(Neutral)**	45	0.0069x	0.7328	-1E-07x^4	0.9522
**pH(Basic)**	15	0.0021x	0.9716	*	*

*: No need because the accuracy is more than 90%

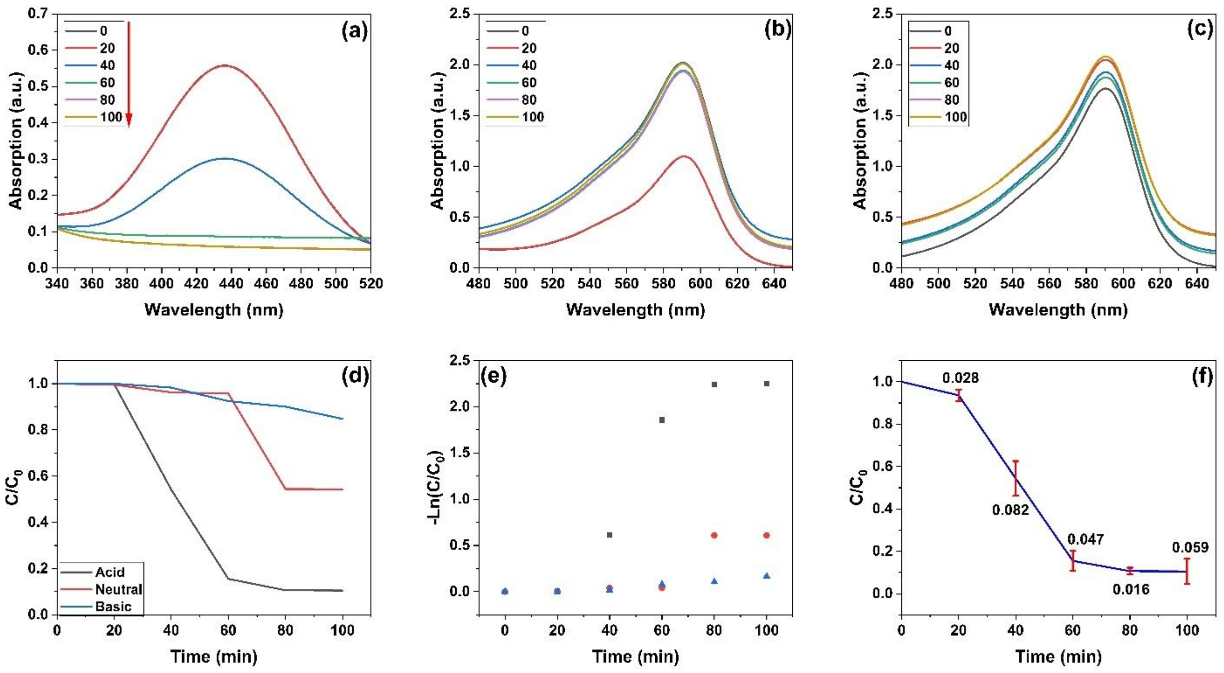

Figure S10. Photocatalytic degradation of bromophenol blue under (a) acidic, (b) neutral, and (c) basic conditions, with (d) efficiency comparison, (e) kinetic plot, and (f) the average of photodecomposition efficiency of NP(Sr) after three runs in an acidic environment with error bars

Table S5. Photocatalytic degradation of methyl orange (MO) by NP(Sr) under visible light, showing kinetic rate constants and correlation coefficients

		First-order rate		Real rate	
	**Degradation (%)**	**K_{app}(min^{-1})**	**R^2**	**K_{app}(min^{-1})**	**R^2**
**pH(acidic)**	60	0.0046x	0.85	3E-06x^3	0.9856
**pH(Neutral)**	30	0.0106x	0.56	-8E-05x^2	0.9932

**pH(Basic)**	15	0.0106x	0.66	9E-06x³	0.9777

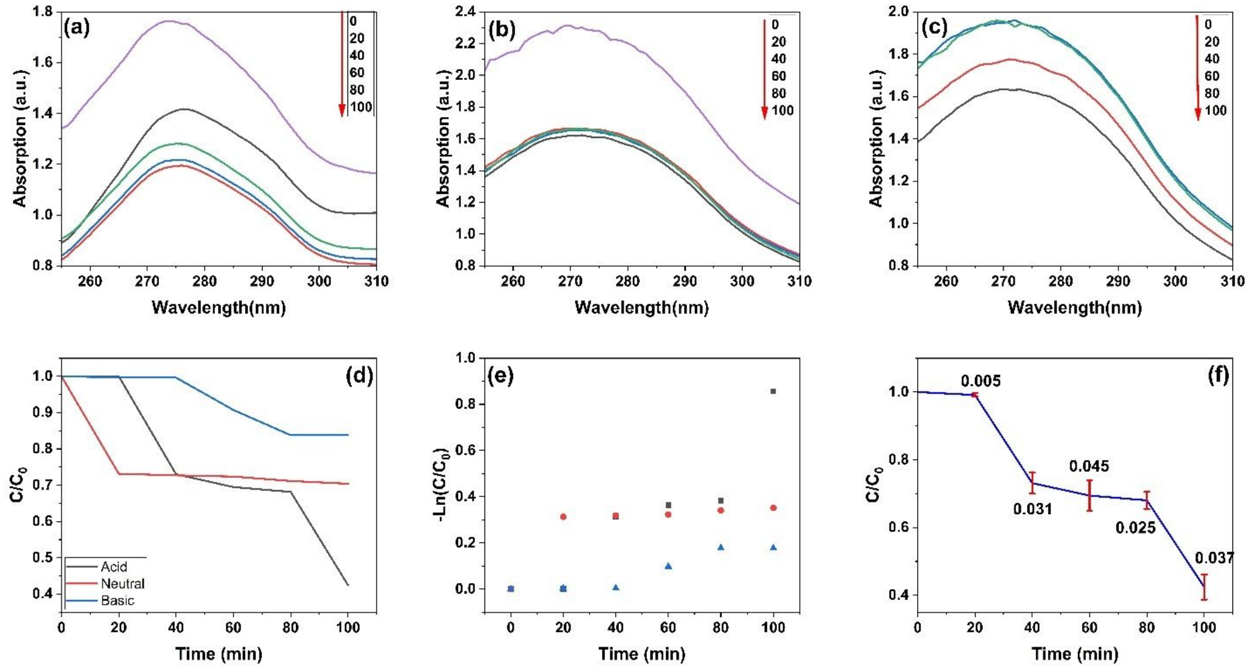

Figure S11. Photocatalytic degradation of methyl orange in (a) acidic, (b) neutral, (c) basic media, with (d) photodecomposition efficiency, (e) kinetic plot, and (f) the average of photodecomposition efficiency of NP(Sr) after three runs in an acidic environment with error bars

Table S6. Degradation kinetics of reactive red 120 (RR120) by NP(Sr) under visible light, listing rate constants and R^2 values.

		First-order rate		Real rate	
	Degradation (%)	$K_{app}(min^{-1})$	R^2	$K_{app}(min^{-1})$	R^2
**pH(acidic)**	100	0.0427x	0.9587	*	*
**pH(Neutral)**	52	0.0075x	0.768	0.0001x²	0.976
**pH(Basic)**	10	0.0006x	0.8657	8E-06x²	0.9861

*: No need because the accuracy is more than 90%

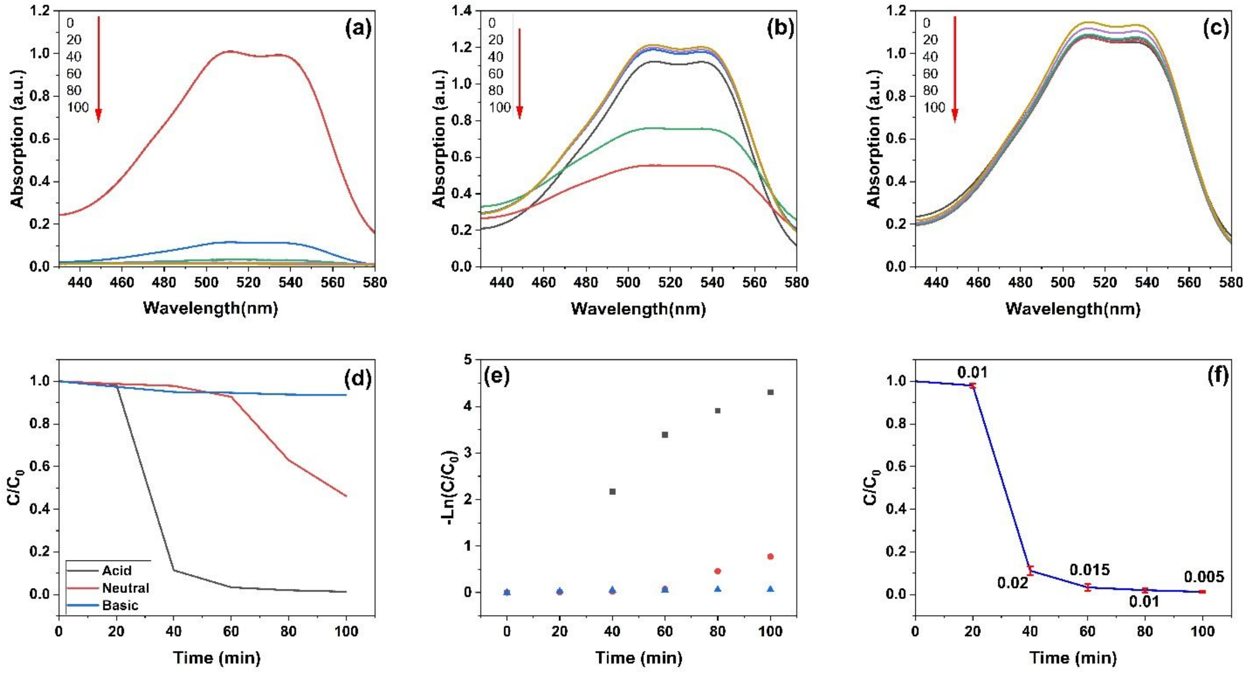

Figure S12. Photocatalytic degradation of reactive red 120 in (a) acidic, (b) neutral, (c) basic environments, showing (d) decomposition efficiency, (e) kinetic plot, and (f) the average of photodecomposition efficiency of NP(Sr) after three runs in an acidic environment with error bars

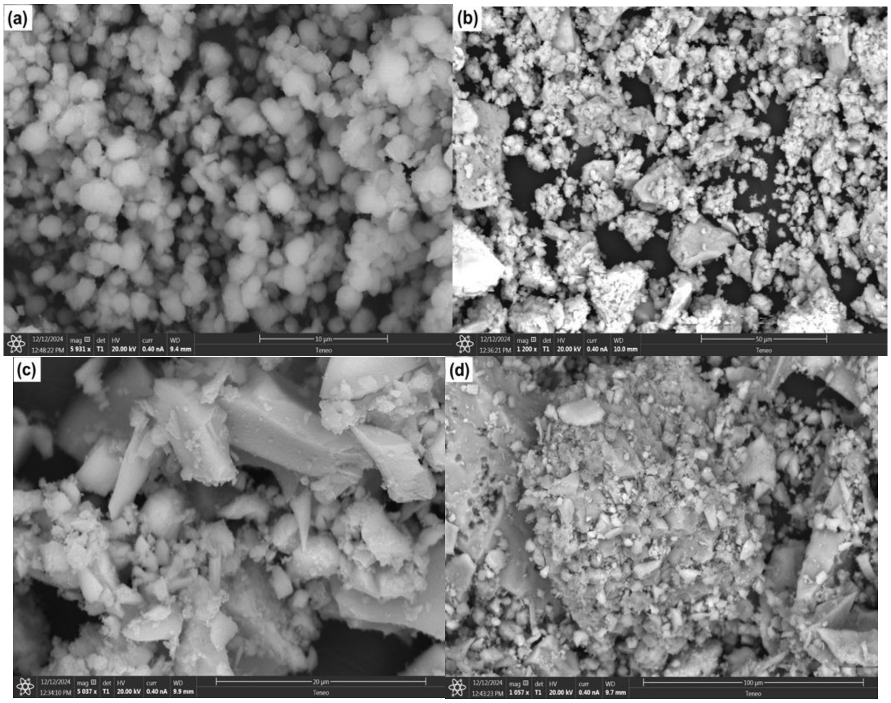

Figure S13. Morphology study of single dopants by FESEM; (a) TiO2, (b) $Ti_{1-y}Ce_yO_{2-\delta}$, (c) $TiO_{2-\delta}N_z$, and (d) $Ti_{1-x}Sr_xO_{2-\delta}$

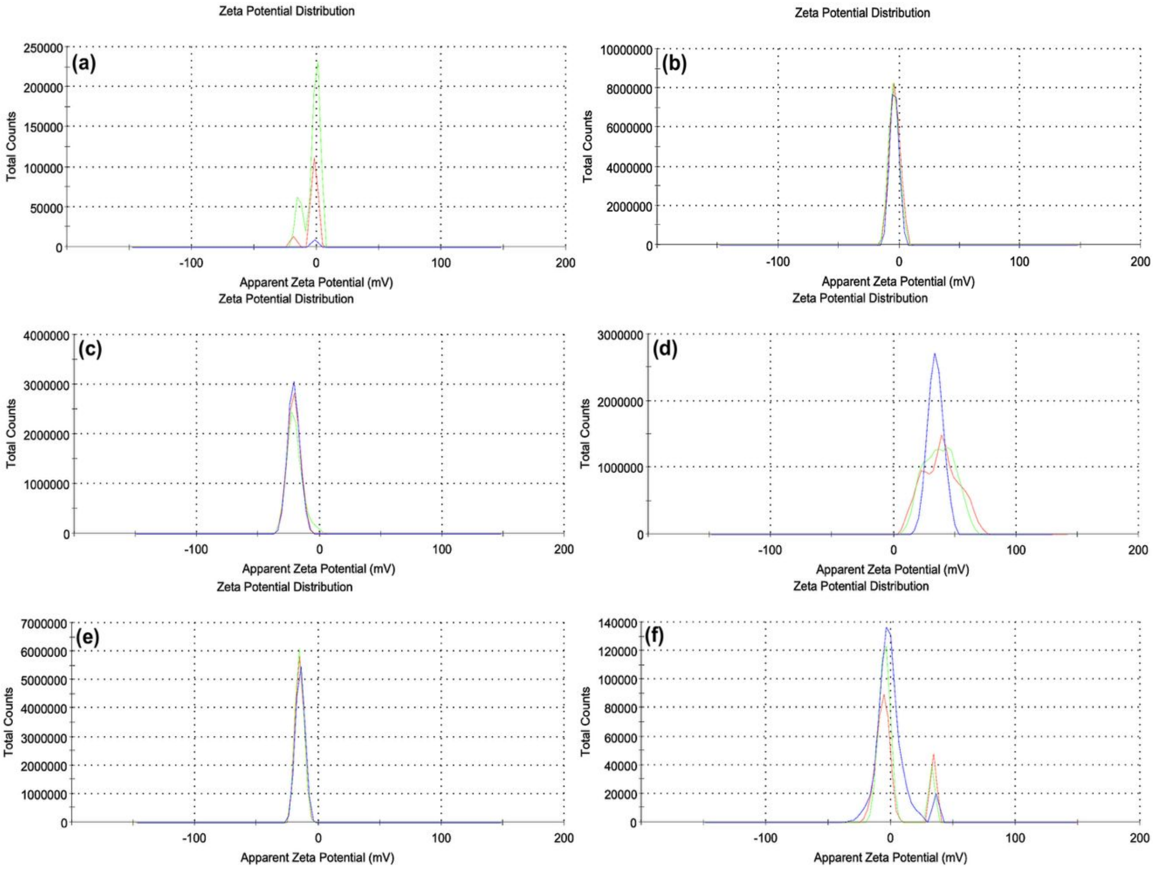

Figure S14. Zeta potential of TiO_2 in (a) acidic (PH 2), (b) neutral, and (c) basic (PH 11), and NP(Sr) in (d) acidic (PH 2), (e) neutral, and (f) basic (PH 11)

Table S7. Coefficients for the natural environment (pH=7)

	Estimate	St. Error	t-value	p-value Pr(>|t|)
**(Intercept)**	63.2941	9.7568	6.487	1.28e-06
**Dye concentration**	38649.0573	20222.0042	1.911	0.0685
**Catalyst dosage**	-402.2857	674.8490	-0.596	0.5569
**Temperature**	0.3255	0.1718	1.894	0.0708

Residual standard error: 10.93 on 23 degrees of freedom
Multiple R-squared: 0.2483, Adjusted R-squared: 0.1502
F-statistic: 2.532 on 3 and 23 DF, p-value: 0.08205

Table S8. Coefficients for the natural environment (pH<7)

	Estimate	St. Error	t-value	p-value Pr(>|t|)
**(Intercept)**	7.674e+01	1.466e+01	5.235	4.01e-05
**Dye concentration**	-3.574e+04	3.225e+04	-1.108	0.281
**Catalyst dosage**	-1.095e+03	1.017e+03	-1.077	0.294
**Temperature**	5.270e-01	2.598e-01	2.028	0.056

Residual standard error: 16.02 on 20 degrees of freedom
Multiple R-squared: 0.2336, Adjusted R-squared: 0.1186
F-statistic: 2.032 on 3 and 20 DF, p-value: 0.1418

Table S9. Coefficients for the natural environment (pH>7)

	Estimate	St. Error	t-value	p-value Pr(>|t|)
**(Intercept)**	99.2330	27.7623	3.574	0.00202
**Dye concentration**	26019.2400	60045.9306	0.433	0.66966
**Catalyst dosage**	145.2043	1887.3621	0.077	0.93948
**Temperature**	-1.4339	0.4934	-2.906	0.00906

Residual standard error: 28.36 on 19 degrees of freedom
Multiple R-squared: 0.313, Adjusted R-squared: 0.2045
F-statistic: 2.885 on 3 and 19 DF, p-value: 0.06262

Table S10. Test of different temperatures (3 repetitions for each)

	pH	Dye concentration (g)	Catalyst dosage (g)	Temp (°C) and repetition	Result	Time to get the final result (min)
**Condition 1**	Basic	0.000166	0.005	30	98%	
				30	98%	80-90 min
				30	100%	
**Condition 2**	Basic	0.000166	0.005	40	99%	
				40	98%	80-90 min
				40	98%	
**Condition 3**	Basic	0.000166	0.005	50	100%	
				50	98%	40-50 min
				50	98%	
**Condition 4**	Basic	0.000166	0.005	60	92%	
				60	91%	120 min
				60	95%	
**Condition 5**	Basic	0.000166	0.005	70	59%	
				70	42%	120 min
				70	53%	

Table S11. Compare the degradation of methylene blue by different multi-dopant photocatalysts under visible light

	**P25**	$Ti_{1-x}Sr_xO_{2-\delta}$	$TiO_{2-\delta}N_z$	$Ti_{1-y}Ce_yO_{2-\delta}$	**NP(Sr)**	$Ti_{1-x}Sr_xO_{2\delta}N_z$	$Ti_{1-y}Ce_yO_{2-\delta}N_z$	$Ti_{1-x-y}Sr_xCe_yO_{2-\delta}$
**Degradation (%)**	6	7	11	17	100	14	23	16
**K_{app}(min^{-1})**	0.0007x	0.0006x	0.0011x	0.0017x	0.0205x	0.0016x	0.0026x	0.0016x
**R^2**	0.9391	0.7759	0.9491	0.786	0.8185	0.9318	0.9346	0.9889

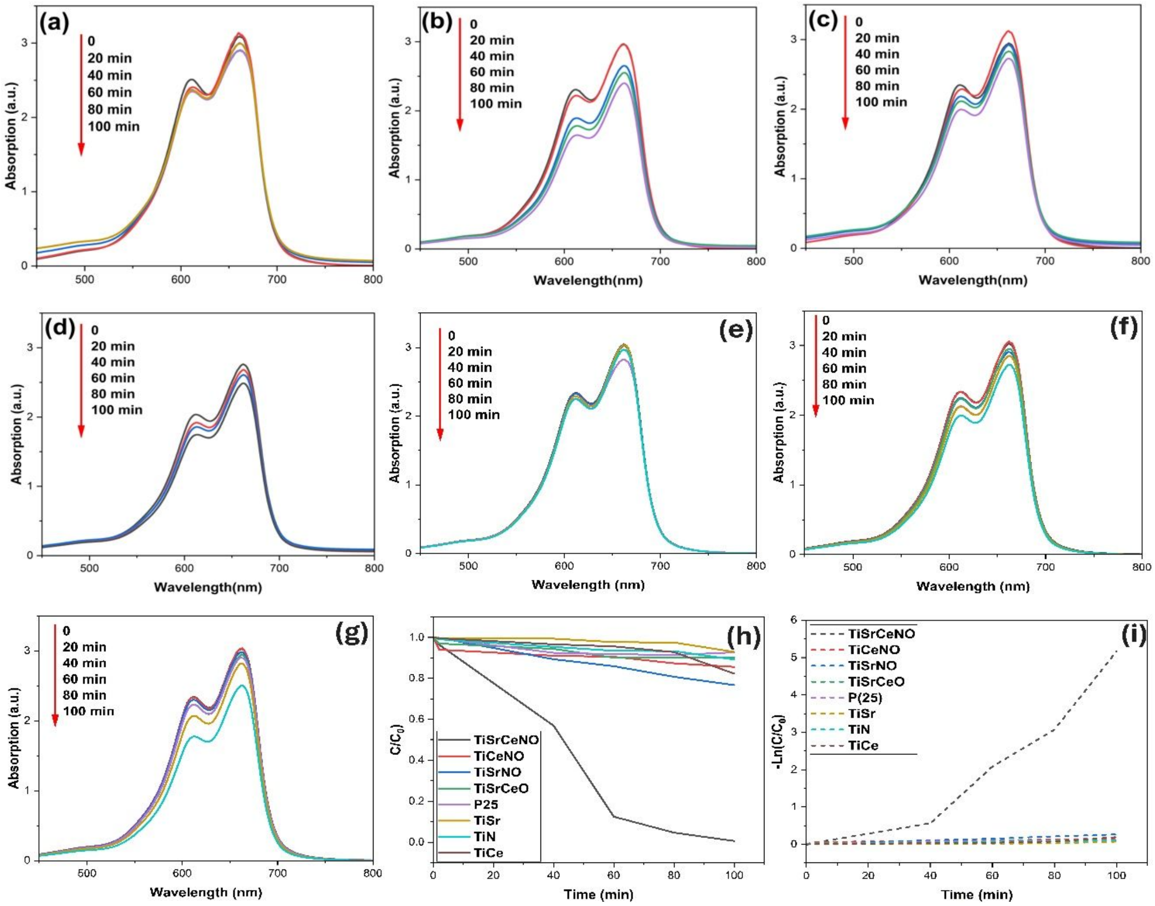

Figure S15. (a) Photocatalytic degradation of methylene blue by (a) TiO_2 (P25), (b) TiSrNO, (c) TiCeNO, (d) TiSrCeO, (e) TiSrO (f) TiNO, (g) TiCeO, (h) photodecomposition efficiency of different multi-doped anatase, and (i) photocatalytic degradation kinetics of the nano-photocatalysts under visible light

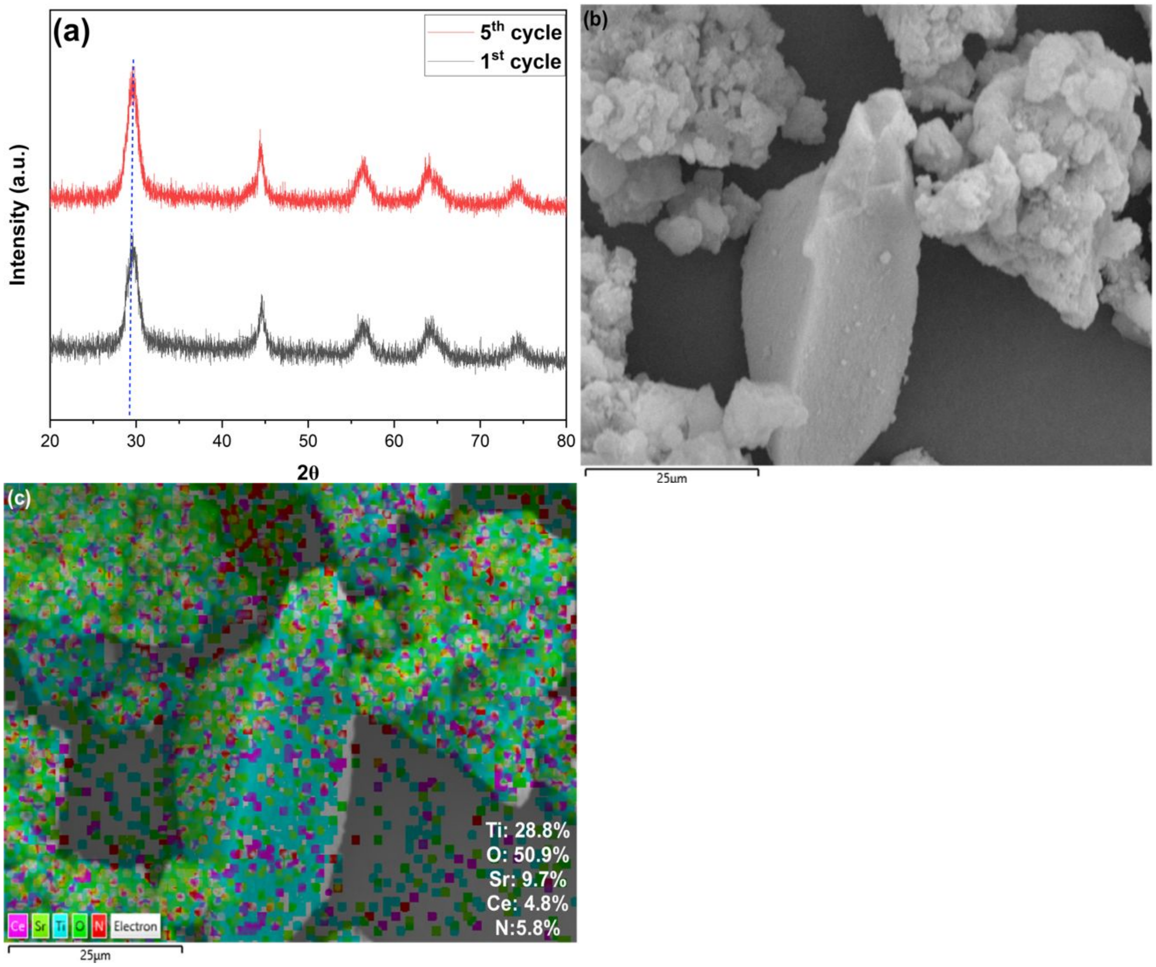

Figure S16. (a) XRD, (b) FESEM, and (C) EDS of the photocatalyst over five consecutive photocatalytic cycles

Table S12. Comparative study of the photocatalytic efficiencies of the different photocatalysts

Catalyst	Reaction	Amount of Catalyst	Conversion (%)	Reaction Time (h)	Source of Light	Temperature (°C)	Ref.
TiO2 (Degussa P25)	Photocatalytic degradation of textile dyes	0.5-2 g/L	80	3	UV	Room Temperature	[1]
ZnO	Photocatalytic degradation of methylene blue	250 mg/L	81	3	UV	Room Temperature	[2]
MgO	Photocatalytic degradation of textile dyes	0.8 g/L	98.3	5	UV	Room Temperature	[3]
SnO2	Photocatalytic degradation of organic dyes	N/A	90	0.83	UV	Room Temperature	[4]
Fe2O3/TiO2	Photocatalytic degradation of Congo red	0.01 w:w	62	N/A	UV	300	[5]
TiO2	Photocatalytic degradation of textile dyes	N/A	>94	6	UV	N/A	[6]
ZnO	Photocatalytic degradation of various dyes	1 g/L	88	1	UV	Room Temperature	[7]
TiO2	Photocatalytic degradation of azo dyes	4 g/L	95	4	UV	475	[8]
Fe2O3	Photocatalytic degradation of Disperse Blue 79	1.96 mg	82	0.67	UV	27	[9]
$SrZrO_3$	Degradation of methylene blue	200 mg	80.1	1.5–2	Visible light	Room temperature	[10]
ZnO	Degradation of methyl orange	N/A	85	1	Simulated solar	20–40	[11]
FVZ3 (Fe-doped ZnO)	Degradation of malachite green	N/A	86	3	UV light	Room temperature	[12]
CuS/PVA	Degradation of rhodamine B	0.25 g	>80	N/A	N/A	Room temperature	[13]
Hypocrellins + H_2O_2	Degradation of rhodamine B	0.18 mM	82.4	1	Visible light	Room temperature	[14]
g-CN	Photocatalytic degradation of caffeine	10 mg	~80	2	Visible Light (420 nm)	Room Temperature	[15]
NP(Sr)	Degradation of different types of textile dyes	7.5 g	~100	2	LED low intensity of visible light	Room temperature	

Table S13. Characteristics of light sources used in photocatalytic studies, including LEDs, xenon lamps, and natural sunlight, showing power, spectral distribution, and intensity.

Light Source	Power (W)	Spectral Distribution	Absolute Intensity (mW/cm^2)	Notes	SPD characteristics	References
LED Light	100	Peaks in the visible spectrum (400-700 nm), customizable for specific wavelengths	5-15	Lower intensity than xenon but more energy-efficient and tunable for specific needs	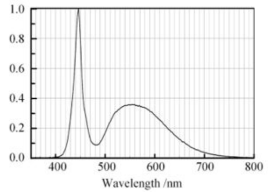	[16]
Xenon Lamp	500	Broad spectrum (UV to near-infrared) with strong UV and IR components	40-50	High intensity across a broad range, but significant energy loss as heat	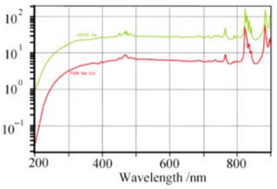	[17]
Natural Sunlight	Variable	Continuous spectrum across UV, visible, and infrared	~1000	Natural and broad-spectrum source, but it varies with time and weather		[18]

Calculating the lamp intensity

The optical and electrical parameters used for estimating the incident light intensity were obtained from the manufacturer's specifications of the employed 300 W LED source. It should be noted that the adopted 25% factor does not represent the intrinsic wall-plug efficiency of the LED device. Instead, it corresponds to an effective radiant power fraction contributing to irradiation at the reaction zone.

Because the 300 W LED lamp is mounted directly above the photocatalytic reactor, illumination occurs predominantly under near-normal incidence with limited geometric obstruction. Under such conditions, only a portion of the electrical input power is effectively delivered as usable optical radiation reaching the reaction medium. Therefore, the adopted factor represents an effective incident irradiation fraction accounting for spectral conversion losses and geometric light utilization within the reactor configuration. This calculation yields an engineering-level estimate for the incident irradiance level without direct radiometric measurement.

The 10 W LEDs are placed laterally around the reactor and shine on the reaction medium at non-normal angles. In this configuration, part of the emitted radiation is reduced due to reflection from reactor walls, angular dispersion, and partial obstruction by reactor components. Consequently, a slightly lower effective radiant contribution (20%) was assumed for the surrounding lamps compared with the directly incident overhead source.

These utilization factors, therefore account for geometric light delivery within the photocatalytic reactor and enable a realistic estimation of incident irradiation intensity when direct optical power measurements are unavailable.

LED Lamp 300W

Lamp Luminous Flux: Φ=45,000 lm
Electrical Power: $P_{electrical}$=300 W
Luminous Efficacy : η= Φ /$P_{electrical}$ =45,000lm/300W=150 lm/W
Radiant Power: 25% efficiency: $P_{radiant}$=$P_{electrical}$×0.25=300 W×0.25=75 W
Distance from Lamp to Beaker: d=0.5 m=50 cm
Box Size: 16.5 in×27 in=41.91 cm×68.58cm
Beam Angle: Since the lamp is mounted on the roof and focused on the beaker, the angle is 90°

Calculate the Illuminated Area of the Beaker
For a 90° beam angle, the light spreads in a circular footprint. The radius of the circle at the beaker's level is:

R=d·tan(θ/2)=50·tan(45°)=50 cm
The illuminated area (A) is:
$A=\pi R^2=\pi(50)^2=7853.98\ cm^2$

Calculate Light Intensity
The light intensity (I) in milliwatts per square centimeter (mW/cm^2) is given by:
$I=P_{radiant}\times1000/A \longrightarrow I=75\times1000/7853.98 = 9.55\ mW/cm^2$

LED Lamp 10W

LED Lamp Parameters
Lamp Luminous Flux: Φ = 1,600 lm
Electrical Power: $P_{electrical}$ = 10 W
Luminous Efficacy: η = Φ / $P_{electrical}$ = 1,600 lm / 10 W = 160 lm/W
Radiant Power: 20 % efficiency → $P_{ra}d_{ia}$□□ = $P_{electrical}$ × 0.20 = 10 W × 0.20 = 2 W
Distance from Lamp to Solution: d = 15 cm
Beam Angle: θ = 120° (lamps positioned around the beaker)

Calculate the Illuminated Area of the Beaker (per lamp)
For a 120° beam, the radius of the light footprint at the beaker's level is:
R = d × tan(θ / 2) = 15 × tan(60°) = 15 × 1.732 = 25.98 cm
The illuminated area (A) is:
$A = \pi R^2 = \pi (25.98)^2 = 2{,}121.6\ cm^2$

Calculate Light Intensity (per lamp)
The light intensity (I) in milliwatts per square centimeter (mW/cm^2) is given by:
$I = (P_{radiant}\times1000)/A \longrightarrow I = (2 \times 1000) / 2{,}121.6 = 0.94\ mW/cm^2$

REFERENCE

(1) Neppolian, B.; Choi, H. C.; Sakthivel, S.; Arabindoo, B.; Murugesan, V. *Solar/UV-Induced Photocatalytic Degradation of Three Commercial Textile Dyes*; 2002; Vol. 89.

(2) Balcha, A.; Yadav, O. P.; Dey, T. Photocatalytic Degradation of Methylene Blue Dye by Zinc Oxide Nanoparticles Obtained from Precipitation and Sol-Gel Methods. *Environmental Science and Pollution Research* **2016**, *23* (24), 25485–25493. https://doi.org/10.1007/s11356-016-7750-6.

(3) Jorfi, S.; Barzegar, G.; Ahmadi, M.; Darvishi Cheshmeh Soltani, R.; Alah Jafarzadeh Haghighifard, N.; Takdastan, A.; Saeedi, R.; Abtahi, M. Enhanced Coagulation-Photocatalytic Treatment of Acid Red 73 Dye and Real Textile Wastewater Using UVA/Synthesized MgO Nanoparticles. *J. Environ. Manage.* **2016**, *177*, 111–118. https://doi.org/10.1016/j.jenvman.2016.04.005.

(4) Li, Y.; Yang, Q.; Wang, Z.; Wang, G.; Zhang, B.; Zhang, Q.; Yang, D. Rapid Fabrication of SnO2 Nanoparticle Photocatalyst: Computational Understanding and Photocatalytic Degradation of Organic Dye. *Inorg. Chem. Front.* **2018**, *5* (12), 3005–3014. https://doi.org/10.1039/c8qi00688a.

(5) Nasirian, M.; Bustillo-Lecompte, C. F.; Mehrvar, M. Photocatalytic Efficiency of Fe2O3/TiO2 for the Degradation of Typical Dyes in Textile Industries: Effects of Calcination Temperature and UV-Assisted Thermal Synthesis. *J. Environ. Manage.* **2017**, *196*, 487–498. https://doi.org/10.1016/J.JENVMAN.2017.03.030.

(6) Khataee, A. R.; Pons, M. N.; Zahraa, O. Photocatalytic Degradation of Three Azo Dyes Using Immobilized TiO2 Nanoparticles on Glass Plates Activated by UV Light Irradiation: Influence of Dye Molecular Structure. *J. Hazard. Mater.* **2009**, *168* (1), 451–457. https://doi.org/10.1016/j.jhazmat.2009.02.052.

(7) Nagajyothi, P. C.; Prabhakar Vattikuti, S. V.; Devarayapalli, K. C.; Yoo, K.; Shim, J.; Sreekanth, T. V. M. Green Synthesis: Photocatalytic Degradation of Textile Dyes Using Metal and Metal Oxide Nanoparticles-Latest Trends and Advancements. *Crit. Rev. Environ. Sci. Technol.* **2020**, *50* (24), 2617–2723. https://doi.org/10.1080/10643389.2019.1705103.

(8) Hachem, C.; Bocquillon, F.; Zahraa, O.; Bouchy, M. Decolourization of Textile Industry Wastewater by the Photocatalytic Degradation Process. *Dyes and Pigments* **2001**, *49* (2), 117–125. https://doi.org/10.1016/S0143-7208(01)00014-6.

(9) Wang, C. T. Photocatalytic Activity of Nanoparticle Gold/Iron Oxide Aerogels for Azo Dye Degradation. *J. Non. Cryst. Solids* **2007**, *353* (11–12), 1126–1133. https://doi.org/10.1016/j.jnoncrysol.2006.12.028.

(10) Tsaviv, J. N.; Eneji, I. S.; Shato'Ato, R.; Ahemen, I.; Jubu, P. R.; Yusof, Y. Photodegradation, Kinetics and Non-Linear Error Functions of Methylene Blue Dye Using SrZrO3 Perovskite Photocatalyst. *Heliyon* **2024**, *10* (14), e34517. https://doi.org/10.1016/j.heliyon.2024.e34517.

(11) Zyoud, A.; Zu'bi, A.; Helal, M. H. S.; Park, D. H.; Campet, G.; Hilal, H. S. Optimizing Photo-Mineralization of Aqueous Methyl Orange by Nano-ZnO Catalyst under Simulated Natural Conditions. *J. Environ. Health Sci. Eng.* **2015**, *13* (1). https://doi.org/10.1186/s40201-015-0204-0.

(12) Mostafa, E. M.; Amdeha, E. Enhanced Photocatalytic Degradation of Malachite Green Dye by Highly Stable Visible-Light-Responsive Fe-Based Tri-Composite Photocatalysts. *Environ. Sci. Pollut. Res. Int.* **2022**, *29* (46), 69861. https://doi.org/10.1007/s11356-022-20745-6.

(13) Al-Kahtani, A. A.; Al-Kahtani, A. A. Photocatalytic Degradation of Rhodamine B Dye in Wastewater Using Gelatin/CuS/PVA Nanocomposites under Solar Light Irradiation. *J. Biomater. Nanobiotechnol.* **2017**, *8* (1), 66–82. https://doi.org/10.4236/jbnb.2017.81005.

(14) Huang, Z.; Zhang, F.; Tang, Y.; Wen, Y.; Wu, Z.; Fang, Z.; Tian, X. Rapid Degradation of Rhodamine B through Visible-Photocatalytic Advanced Oxidation Using Self-Degradable Natural Perylene Quinone Derivatives—Hypocrellins. *Bioengineering 2022, Vol. 9, Page 307* **2022**, *9* (7), 307. https://doi.org/10.3390/bioengineering9070307.

(15) Liu, H.; Wang, C.; Wang, G. Photocatalytic Advanced Oxidation Processes for Water Treatment: Recent Advances and Perspective. *Chem. Asian J.* **2020**, *15* (20), 3239–3253. https://doi.org/10.1002/asia.202000895.

(16) Lukovic, M.; Lukovic, V.; Belca, I.; Kasalica, B.; Stanimirovic, I.; Vicic, M. LED-Based Vis-NIR Spectrally Tunable Light Source - the Optimization Algorithm. *Journal of the European Optical Society* **2016**, *12* (1), 1–12. https://doi.org/10.1186/S41476-016-0021-9/FIGURES/12.

(17) Zhang, Z.; Lu, R.; Zhang, A.; Li, H.; Liu, J.; Zhang, Z.; Lu, R.; Zhang, A.; Li, H.; Liu, J. Monochromatic LED-Based Spectrally Tunable Light Source for Chromatic Confocal Sensors. *OptEn* **2023**, *62* (02), 024102. https://doi.org/10.1117/1.OE.62.2.024102.

(18) Yang, P.; Wu, Z.; Chen, Y.; Guo, Y.; Zhang, P.; Song, H. Spectral Irradiance Distribution in Underwater Light Field Generated by an LED Light Source. *J. Coast. Res.* **2020**, *103* (sp1), 453–457. https://doi.org/10.2112/SI103-092.1.
